# 16S rRNA gene sequencing and healthy reference ranges for 28 clinically relevant microbial taxa from the human gut microbiome

**DOI:** 10.1371/journal.pone.0176555

**Published:** 2017-05-03

**Authors:** Daniel E. Almonacid, Laurens Kraal, Francisco J. Ossandon, Yelena V. Budovskaya, Juan Pablo Cardenas, Elisabeth M. Bik, Audrey D. Goddard, Jessica Richman, Zachary S. Apte

**Affiliations:** 1 uBiome, Inc., San Francisco, California, United States of America; 2 Department of Biochemistry and Biophysics, University of California, San Francisco, San Francisco, California, United States of America; Texas A&M University College Station, UNITED STATES

## Abstract

Changes in the relative abundances of many intestinal microorganisms, both those that naturally occur in the human gut microbiome and those that are considered pathogens, have been associated with a range of diseases. To more accurately diagnose health conditions, medical practitioners could benefit from a molecular, culture-independent assay for the quantification of these microorganisms in the context of a healthy reference range. Here we present the targeted sequencing of the microbial 16S rRNA gene of clinically relevant gut microorganisms as a method to provide a gut screening test that could assist in the clinical diagnosis of certain health conditions. We evaluated the possibility of detecting 46 clinical prokaryotic targets in the human gut, 28 of which could be identified with high precision and sensitivity by a bioinformatics pipeline that includes sequence analysis and taxonomic annotation. These targets included 20 commensal, 3 beneficial (probiotic), and 5 pathogenic intestinal microbial taxa. Using stool microbiome samples from a cohort of 897 healthy individuals, we established a reference range defining clinically relevant relative levels for each of the 28 targets. Our assay quantifies 28 targets in the context of a healthy reference range and correctly reflected 38/38 verification samples of real and synthetic stool material containing known gut pathogens. Thus, we have established a method to determine microbiome composition with a focus on clinically relevant taxa, which has the potential to contribute to patient diagnosis, treatment, and monitoring. More broadly, our method can facilitate epidemiological studies of the microbiome as it relates to overall human health and disease.

## Introduction

The human gut microbiota, the consortium of microbial inhabitants in our distal gut, has been increasingly recognized as playing a major role in the maintenance, promotion and distortion of health. A healthy gut microbiota is involved in energy extraction from dietary components [1,2], regulation of components of the immune system [3], vitamin synthesis [4], and colonization resistance, i.e., protection against colonization by gastrointestinal pathogens [5]. In addition, there is an increasing number of associations between a microbiome imbalance and various diseases and medical conditions [6]. Such disturbances of the healthy microbiome composition have been found associated with infections with gastrointestinal pathogens such as *Campylobacter*, *Salmonella* and *Vibrio cholerae* [7,8] to more elusive imbalances found in the setting of inflammatory bowel diseases [9,10], metabolic syndrome [11], and irritable bowel syndrome [12,13].

Rapid and accurate identification of pathogens is critical to provide the appropriate treatment for patients suffering from certain gastrointestinal conditions. This has in particular been the case for acute diarrheal illness, for which identification of the causative agents still greatly relies on conventional microbiology techniques such as culturing of stool samples [14]. However, although culture-based methods are rapid, sensitive, and specific, they are often designed around a presence/absence criterion, i.e., to detect microbial organisms that are usually absent in health and present in disease. Traditional clinical microbiology methods are less able to detect potential gut microbiota imbalances, i.e. aberrant ratios of multiple non-pathogenic, health-associated microorganisms in the setting of chronic conditions. One of the main reasons is that most intestinal commensals are hard to culture and can only be recovered under specialized technical conditions [15]. Recent advancements in amplification and next-generation sequencing (NGS) techniques, in particular applied to the bacterial and archaeal ribosomal RNA encoding genes (16S rRNA genes) have overcome this problem, are increasingly used in the clinical microbiology lab [16,17], and have enormously expanded our knowledge of microbiome composition [18].

However, it is still difficult to use the composition of the human gut microbiota as a clinical tool in the diagnosis of chronic health conditions. This is partly caused by large inter-individual variations associated with human geographic, dietary, genetic and lifestyle differences, which made it challenging to define the healthy human microbiome [19,20]. Therefore, most studies comparing microbiomes from healthy controls and diseased patients might be too small to detect small, but real, differences in gut microbiotas.

**Fig 1:**
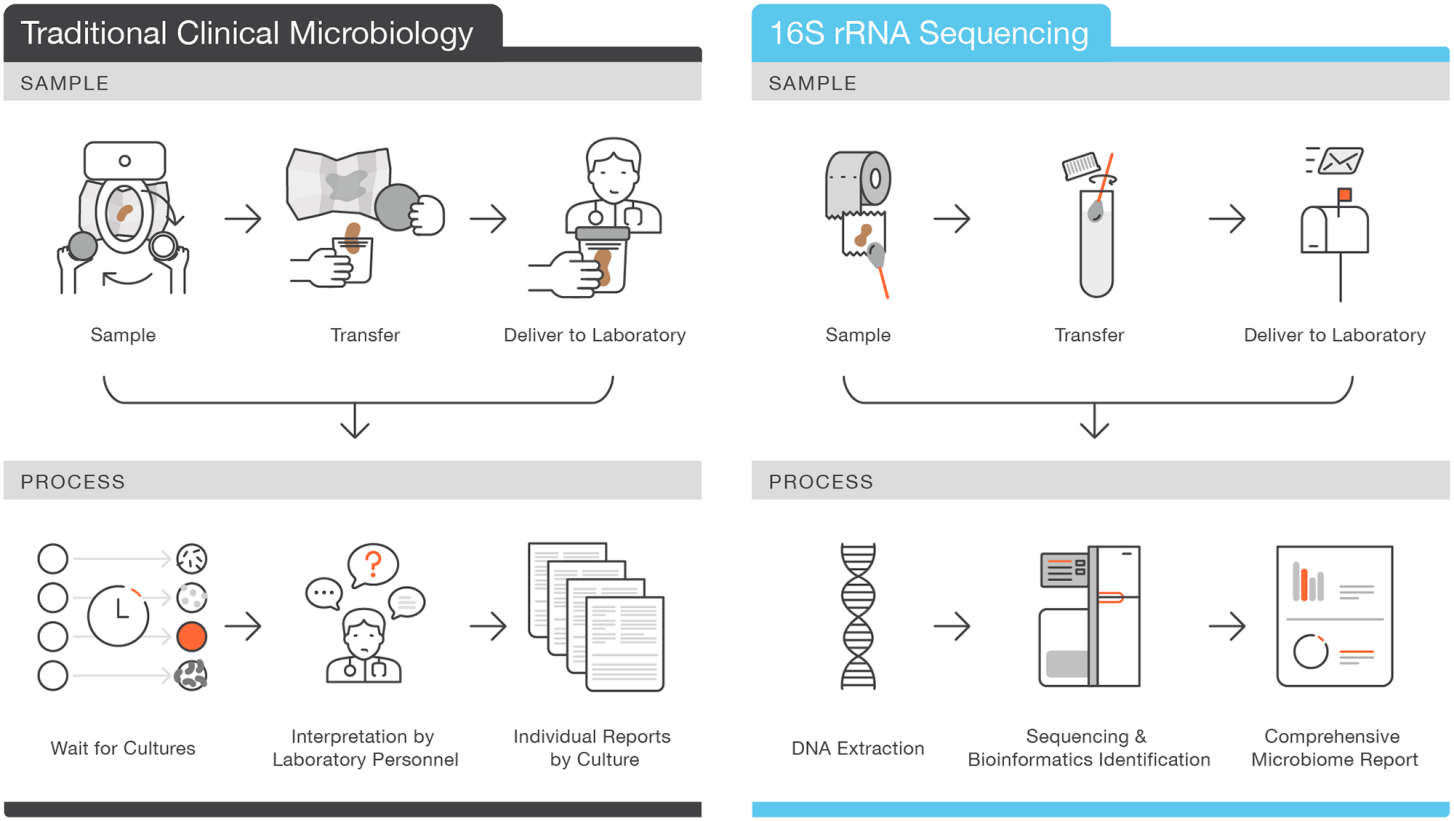
Sample collection and processing of clinical stool samples for traditional clinical microbiology versus 16S rRNA gene sequencing. A traditional fecal microbiology test requires collecting a rather large stool sample in a cumbersome process and immediately delivery to the laboratory or clinical practitioner. Specific organisms are cultured from the sample based on the physician’s requests, and processing requires interpretation by extensively trained laboratory personnel. This approach usually focuses on the discovery of culturable pathogens. In contrast, 16S rRNA gene sequencing requires only a fraction of the biological material needed for culture-based techniques (just a swab from toilet paper). In addition, the sample is collected in tube with a buffer that lyses microorganisms and stabilizes DNA, allowing the sample to be mailed at room temperature. Thus, sample collection and delivery are greatly simplified. Sequencing and interpretation can be automated to reduce human labor and error. Finally, this method can detect uncultivable organisms and relative abundances of both pathogenic and commensal organisms.

In this study, we present an NGS-based clinical gut microbiome sequencing assay to assess the relative abundance of health condition-associated microorganisms (Fig 1). The assay utilizes 16S rRNA gene sequencing to identify 28 clinically relevant microbial targets (14 species and 14 genera), including 5 intestinal pathogens, 3 beneficial bacteria, and 20 commonly present inhabitants of the human gastrointestinal tract, with high precision and sensitivity. In addition, we define the relative abundance ranges of these taxa in stool samples from a large healthy human cohort.

## Material and methods

### Participants

A group of 1,000 self-reported healthy individuals who had submitted fecal samples (one sample per subject) were selected from the ongoing uBiome citizen science microbiome research study (manuscript in preparation). Of these, 103 extracted fecal samples (see below for more details) did not pass our 10,000 read quality control threshold. We used this stringent threshold to ensure detection of all targeted taxa, even at low abundance. The final cohort therefore included 897 individuals (62% male and 38% female). Participants were explicitly asked about 42 different medical conditions such as cancer, infections, obesity, chronic health issues, and mental health disorders. Selected participants with an average age of 39.7 years (SD = 15.5) responded to an extensive survey and self-reported to be currently and overall in good health. None of the individuals selected for the healthy cohort had ever been diagnosed with high blood sugar, diabetes, gut-related symptoms, or any other medical condition. This study was performed under a Human Subjects Protocol provided by an IRB (E&I Review Services, IRB Study #13044, 05/10/2013). Informed consent was obtained from all participants. Analysis of participant data was performed in aggregate and anonymously.

### Sample collection and 16S rRNA gene sequencing

Fecal samples were self-collected by participants at home using commercially available uBiome microbiome sampling kits, which have been designed to follow the specifications laid out by the NIH Human Microbiome Project [21]. Participants were instructed to use a sterile swab to transfer a small amount of fecal material into a vial containing a lysis and stabilization buffer that preserves the DNA for transport at ambient temperatures. Samples were lysed using bead-beating, and DNA was extracted in a class 1000 clean room by a guanidine thiocyanate silica column-based purification method using a liquid-handling robot [22, 23]. PCR amplification of the 16S rRNA genes was performed with primers containing universal primers amplifying the V4 variable region (515F: GTGCCAGCMGCCGCGGTAA and 806R: GGACTACHVGGGTWTCTAAT) [24]. In addition, the primers contained Illumina tags and barcodes. Samples were barcoded with a unique combination of forward and reverse indexes allowing for simultaneous processing of multiple samples. PCR products were pooled, column-purified, and size-selected through microfluidic DNA fractionation [25]. Consolidated libraries were quantified by quantitative real-time PCR using the Kapa Bio-Rad iCycler qPCR kit on a BioRad MyiQ before loading into the sequencer. Sequencing was performed in a pair-end modality on the Illumina NextSeq 500 platform rendering 2 x 150 bp pair-end sequences.

### Taxonomic annotation and reference database generation

After sequencing, demultiplexing of samples was performed using Illumina's BCL2FASTQ algorithm. Reads were filtered using an average Q-score > 30. Forward and reverse reads were appended together after removal of primers and any leading bases, and clustered using version 2.1.5 of the Swarm algorithm [26] using a distance of 1 nucleotide and the “fastidious” and “usearch-abundance” flags. The most abundant sequence per cluster was considered the real biological sequence and was assigned the count of all reads in the cluster. The remainder of the reads in a cluster were considered to contain errors as a product of sequencing. The representative reads from all clusters were subjected to chimera removal using the VSEARCH algorithm [27]. Reads passing all above filters (filtered reads) were aligned using 100% identity over 100% of the length against a hand-curated database of target 16S rRNA gene sequences and taxonomic annotations derived from version 123 of the SILVA database [28]. The hand-curated databases for each taxa were created by selectively removing sequences with amplicons that were ambiguously annotated to more than one taxonomic group, while still maximizing the performance metrics sensitivity, specificity, precision, and negative predictive value of identification for the remaining amplicons in each taxa (S1 Doc). In total 28 taxonomic groups of clinical relevance passed our criteria of over 90% for each performance metric (S1 Table). Raw FASTQ reads mapping to the samples and the taxa in the reference databases used in this study were uploaded to EBI’s ENA under accession code PRJEB20022. The relative abundance of each taxa was determined by dividing the count linked to that taxa by the total number of filtered reads.

### Experimental verification

Verification samples were obtained from Luminex‘s xTAG Gastrointestinal Pathogen Panel (xTAG GPP). Verification samples contained real or synthetic stool samples with live or recombinant material, with some specimens being positive for more than one clinical target. A total of 40 positive control samples were used, 35 of which were certified to be positive for one control taxon from our target list, with the exception of those samples containing either *Clostridium difficile* or *Salmonella enterica* which are positive for 2 taxa simultaneously (the species to which they belong and their corresponding genus). The control samples were considered negative for the remainder of the taxa on our test panel. Two out of 35 control samples did not pass our sequencing quality thresholds of having at least 10,000 pair-end reads each, so they were removed from further analysis. Five additional Luminex samples positive for *Yersinia*, a genus that is not present in the final target list, were included as negative controls. Verification samples were processed in uBiome microbiome sampling kits using the clinical pipeline described above.

## Results and discussion

### Clinically relevant target identification

To derive a preliminary target list of bacteria and archaea to include in our assay, we first identified clinically relevant microorganisms present in the human microbiome. We performed an extensive review of the literature and clinical landscape, and obtained evidence supporting the importance of hundreds of microorganisms known to inhabit the human gut. We included these in our initial list, along with organisms that are commonly interrogated in clinical tests. This initial list was further evaluated for positive and negative associations with several indications, including flatulence, bloating, diarrhea, gastroenteritis, indigestion, abdominal pain, constipation, infection, inflammatory bowel syndrome, ulcerative colitis, and Crohn's disease-related conditions. Ultimately, we compiled a preliminary target list containing 15 genera and 31 species of microorganisms associated with human health status (S1 Table), including pathogenic, commensal, and probiotic bacteria and archaea.

**Fig 2:**
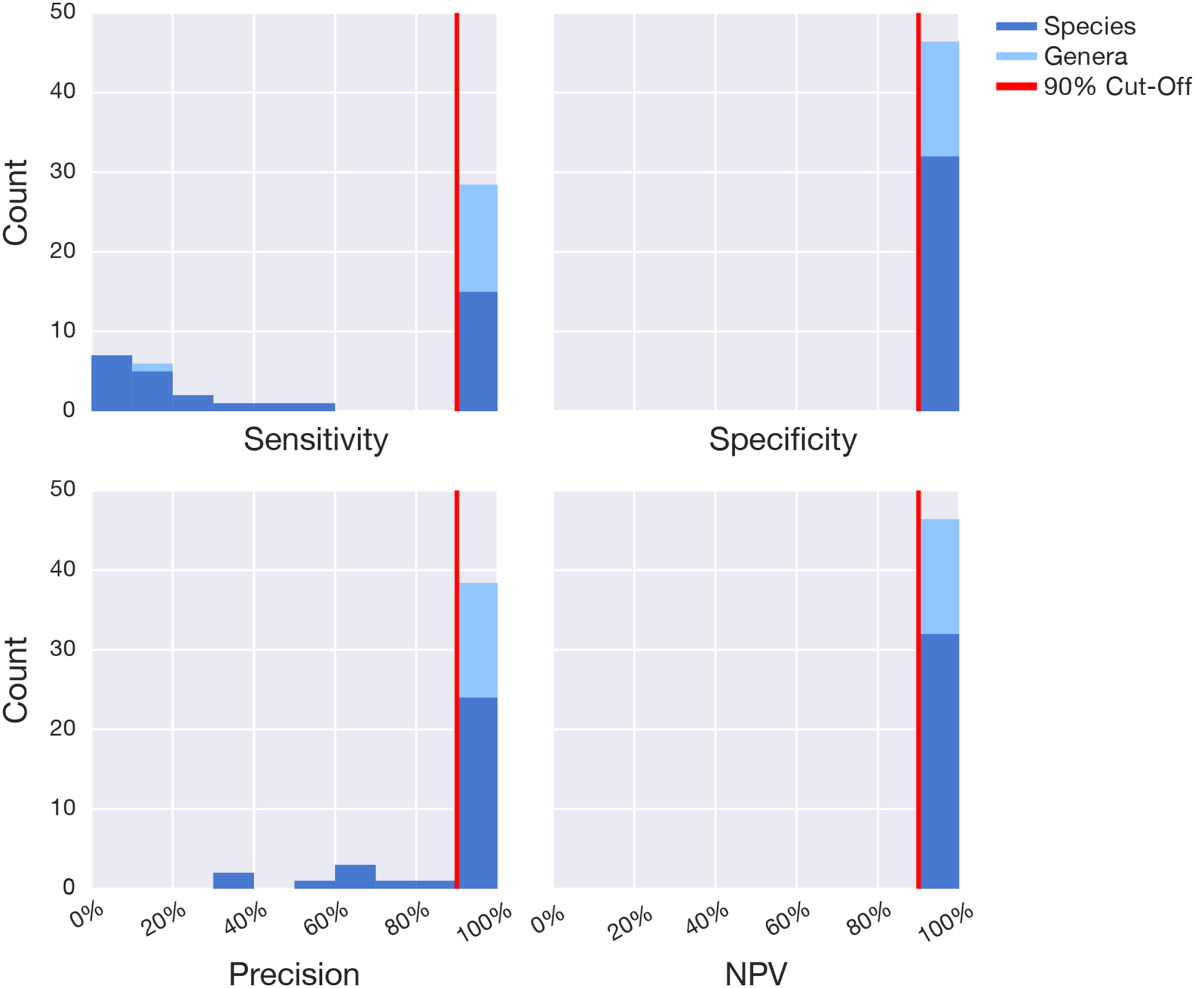
Bioinformatics target identification performance metrics. The 46 preliminary targets identified from literature and available clinical tests are comprised of 15 genera and 31 species. To optimize the bioinformatics pipeline for accurate detection of the maximum number of targets, the following performance metrics were evaluated based on the number of true positives (TP), true negatives (TN), false positives (FP), and false negatives (FN) detected in a manually curated amplicon database (described in S1 Doc): specificity = TN / (TN + FP); sensitivity = TP / (TP + FN); precision = TP / (TP + FP); and negative predictive value (NPV) = TN / (TN + FN). After optimization, 28/46 preliminary targets passed our stringent threshold of 90% (red vertical line) for each of the parameters, resulting in the accurate detection of all genera (light blue) except for *Pseudoflavonifractor*, and 14/31 species (dark blue).

The bioinformatics annotation pipeline developed for this method was specifically designed to have high prediction performance. To this end, we implemented a taxonomy annotation based on sequence searches of 100% identity over the entire length of the 16S rRNA gene V4 region from the preliminary targets in our database (S1 Doc). Curated databases were generated for each of the taxa in our preliminary target list using the performance metrics sensitivity, specificity, precision, and negative predictive value as optimizing parameters. In other words, the bioinformatics pipeline was optimized to ensure that a positive result truly means the target is present in the sample and a negative result is only obtained when no target is present in the sample. After optimizing the confusion matrices for all preliminary targets, 28 out of 46 targets passed our stringent threshold of 90% for each of the parameters (Fig 2). The resulting target list is composed of 5 known pathogens, 3 beneficial bacteria, and 20 additional microorganisms related to various gut afflictions (S2 Table), including commensal bacteria and one archaeon. On average the sensitivity, specificity, precision, and negative prediction value of the microorganisms on our target list are 99.0%, 100%, 98.9%, and 100%, for the species, and 97.4%, 100%, 98.5%, and 100% for the genera.

### Reference ranges from a healthy cohort

**Fig 3:**
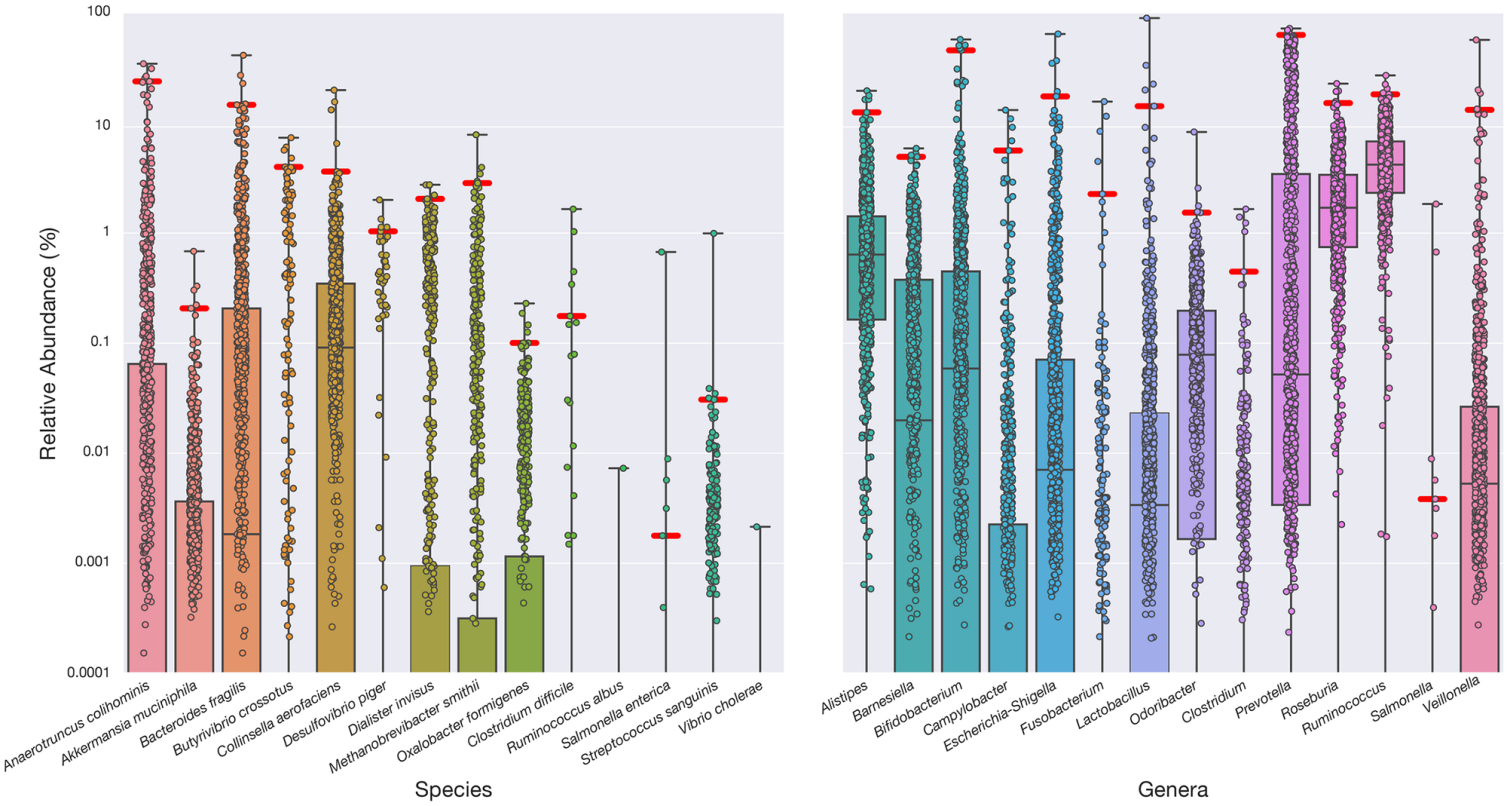
Reference ranges from a cohort of healthy individuals for 28 clinically relevant species and genera. Healthy participant stool microbiome data were analyzed to determine the empirical reference ranges for each target. The boxplot displays the relative abundance for each of 897 self-reported healthy individuals, revealing the healthy ranges of abundance for the taxa in the test panel. The healthy distribution is used to define the 99% confidence interval (red line). Boxes indicate the 25th–75th percentile, and the median coverage is indicated by a horizontal line in each box. Even in this healthy cohort, many of the bacteria that are associated with poor health conditions are present at some level. As most taxa are absent in a significant number of individuals most boxes expand to 0%, the healthy lower limit (not shown).

Many clinically relevant microorganisms associated with health and disease are present at some level in the gut of healthy individuals. The clinical significance of microbiome test results is determined not only by the identity, but also the quantity of distinct species and genera within the context of a healthy reference range. To determine the healthy reference range for the 28 targets, we established a cohort of 897 samples from self-reported healthy individuals from the uBiome microbiome research study (manuscript in preparation). Microbiome data from this cohort were analyzed to determine the empirical reference ranges for the 14 species and 14 genera. For each of the 897 samples, we determined the relative abundance of each target within the microbial population. This analysis gave rise to a distribution of relative abundance for each target in the cohort (Fig 3, S3 Table). These data were used to define a central 99% healthy range with confidence intervals for each target. Many of the targets show significant spread, emphasizing the importance of microbiome identification in the context of a reference range. For example, the pathogen *C*. *difficile* is found in ~2% of the healthy cohort, and thus we define a healthy range for it from 0% to 0.18% relative abundance. Although *C*. *difficile* is an opportunistic pathogen that can cause severe diarrhea, especially among antibiotic-treated hospitalized patients [29], our results confirm that asymptomatic *C*. *difficile* colonization is not uncommon in healthy individuals [30]. Although all taxa were present in at least one of the healthy individuals, the upper limit of the reference range of the relative abundance was found to be quite high for some taxa (e.g., 63% for *Prevotella* and 49% for *Bifidobacterium*). Two species are not represented at all within the central 99% of the healthy cohort: *Vibrio cholerae* and *Ruminococcus albus*. The absence of *V*. *cholerae* is suggestive of its pathogenic nature and its relatively rare occurrence in the developed world. However, *R*. *albus*, has previously been found to be enriched in healthy subjects in comparison to patients with Crohn’s disease [31].

### Detection of known pathogens above the healthy reference range

**Fig 4:**
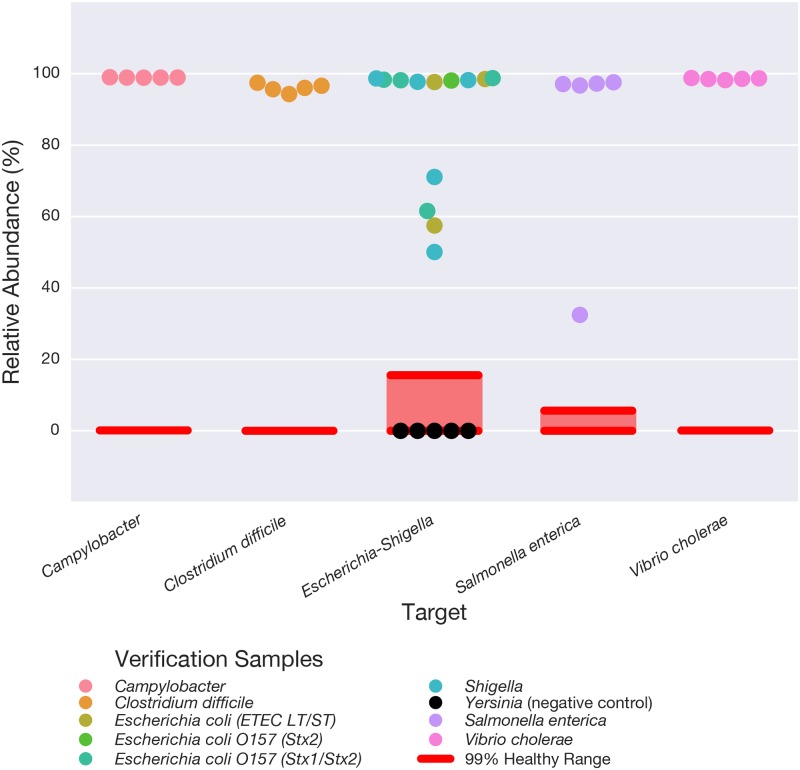
Experimental validation of the clinical 16S rRNA gene sequencing for pathogens on the screening test panel using verification samples. Commercially available verification samples (Luminex) containing real or synthetic stool samples positive for at least one control taxon from the target panel were tested using the DNA extraction, amplification and bioinformatics pipeline described in this paper. Of the 35 samples on this panel, 33 yielded 10,000 or more reads. Together, these 33 samples contained the 5 pathogenic taxa in our target list, all of which were accurately identified at a level above the maximum value of the healthy range (red lines). All 33 control samples tested within the healthy range for the remainder of the taxa on our panel (not shown), and thus were considered negative for the pathogenic taxa shown here. Five samples positive for *Yersinia*, a genus that is not present in our target list, were included as additional negative controls. These samples are visualized for the *Escherichia-Shigella* genus as they contained DNA for this taxon within the healthy range.

After establishing our ability to detect all 28 targets using synthetic DNA at relative abundances of 0.03% or more (S2 Doc, S4 Table), we tested 40 reference isolates from Luminex’s xTAG Gastrointestinal Pathogen Panel to establish the clinical relevance of our pipeline. These verification samples comprise real or synthetic stool samples with live or recombinant material of known composition. Two of the samples were excluded due to poor sequencing depth. The remaining samples were positive for 1 of 8 different bacterial strains corresponding to 5 of our clinical targets: *V*. *cholerae* (5), *S*. *enterica* (5), *Escherichia-Shigella* (13), *Campylobacter* (5) and *C*. *difficile* (5). All of these verification samples were correctly identified as having a relative abundance of the clinical target well above our defined healthy reference range (Fig 4). Five samples containing *Yersinia* were tested as a negative control. Although *Yersinia* was included in our preliminary target list, it did not pass our stringent bioinformatics QC thresholds for accurate identification. As expected, the relative abundance of the 28 clinical targets was in the healthy range for the *Yersinia* positive samples, as shown for *Escherichia-Shigella* (Fig 4).

### Clinical relevance

**Fig 5:**
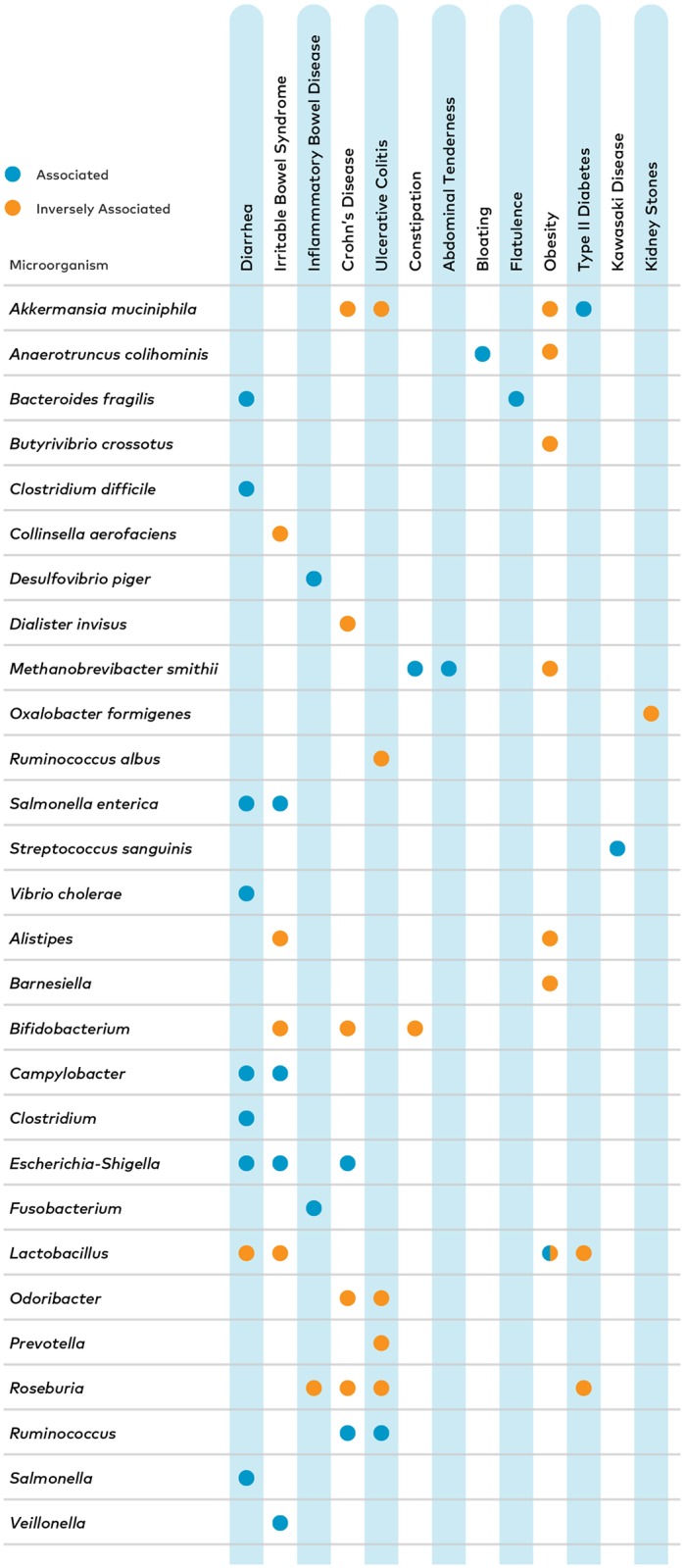
Human health associations of the 28 targets microorganisms. All of the 28 taxa on the test have been associated with human health in the gut microbiome. Here we show the associations for 13 specific conditions. 13 of the taxa are associated with health conditions, meaning that these microorganisms have been shown to be elevated in patients suffering from these conditions. The 11 microorganisms that are inversely associated were found to be less abundant in people who have this condition in the scientific literature (S2 Table). 4 taxa are associated with some and inversely associated with other conditions. Interestingly, both elevated and reduced levels of *Lactobacillus* have been associated with obesity [44–46].

Accurate detection of microorganisms in the context of a healthy reference range can be of great use to physicians. All of the 28 microorganisms successfully identified using 16S rRNA gene sequencing are associated with specific health conditions. For example, 2 of the microorganisms on our panel, *Escherichia-Shigella* and *Ruminococcus*, are associated with Crohn’s disease [32–37], while 5 other organisms, *Akkermansia muciniphila*, *Bifidobacterium*, *Dialister invisus*, *Odoribacter and Roseburia*, are inversely associated with Crohn’s disease [32,35–38] (Fig 5, S2 Table). To help diagnose and monitor this condition and distinguish it from other conditions with other microbial associations, it is essential to sequence a panel of microorganisms. The combinatorial information of which organisms are outside of the healthy range can be used by a physician to augment a treatment plan. For example, reducing the intake of animal based diets and diets high in resistant starches to reduce *Ruminococcus* [39–41] and the consumption of probiotics, inulin and oligofructoses to increase levels of *Bifidobacterium* [42,43].

The accurate detection of a great number of microorganisms within a stool sample is critical to initiate the appropriate treatment in a clinical setting. Here we have shown that 16S rRNA gene sequencing can accurately detect and quantify clinically relevant levels of 28 target bacteria and archaea. We demonstrate that many prokaryotic targets identified from the literature as associated with human health can be consolidated in an assay, and further that relating the relative levels of bacteria and archaea to a healthy reference range enables the reporting of positive results only when clinically relevant.

The selection of microorganisms for this panel was based on studies in medical journals and peer-reviewed articles. While all targets are relevant on their own, there is some overlap in the consolidated test. For example, while the *Salmonella* genus is unquestionably clinically relevant, testing for the genus when the test already includes the *Salmonella enterica* species might be clinically redundant. The only other species of *Salmonella* is *Salmonella bongori*, a species that rarely infects humans and is mostly relevant to lizards [47]. In our dataset of nearly 900 stool samples from healthy individuals, eight samples tested positive for the genus-level *Salmonella* target (S3 Table). In 6 of these, the relative *Salmonella*-genus abundance was less than 0.01%, the clinical relevance of which remains unclear. In one of the two remaining subjects, both *Salmonella*-genus and *S*. *enterica* abundance values were 0.674%, suggesting the same target was detected. In the remaining sample, *Salmonella-genus* was present at 1.84% but *S*. *enterica* was not detected, suggesting that this individual might have been colonized with *S*. *bongori*. Of note, none of these individuals reported having gastrointestinal problems. It remains to be determined whether these low counts of Salmonella are suggestive of the presence of clinically irrelevant, yet-uncharacterized strains, as has been reported in cattle [48].

While medical diagnosis has traditionally been focused on pathogens, research on the whole microbiome and its correlations with gut health continues to emerge [6,20]. The test panel presented here reports on some microorganisms that are not usually interrogated in the clinic but provide additional insight into the overall gut health of a patient in a clinical setting (S2 Table). Because our detection method is based on DNA sequencing, the target panel can readily be expanded if new information about clinically important microorganisms arises. Because 16S rRNA gene sequencing identifies and quantifies the bacteria and archaea in a sample, relevant microbial metrics such as a microbiome diversity score can also be obtained, in addition to the information about individual targets, to provide a comprehensive overview of gastrointestinal health [49,50].

As any rRNA gene based test, this assay has limitations. The test only detects and analyzes a short, specific genomic region, and taxonomic resolution or functional inference is therefore limited. For example, this assay cannot recognize the different serovars within *S*. *enterica*, or detect toxin genes that could distinguish pathogenic *C*. *difficile* or *Escherichia* strains from nonpathogenic strains, or resolve species within some of the genus-level targets. The correlation—or lack thereof—of 16S rRNA-based phylogenetic sequence identities with taxonomic levels such as genus or species has been extensively discussed elsewhere [51–54].

16S rRNA gene sequencing as a clinical screening tool for gut-related conditions has many advantages over traditional culture-based techniques, including ease of sampling, scalability of the test, no need for human interpretation, and the ability to provide additional information about gut health. Most importantly, it can determine the relative abundances of multiple microbial targets, and can therefore be used to detect potential deviations of one or many taxa from that of a healthy cohort. Defining the healthy ranges for gut microbes with known clinical relevance, as done in this study, is likely to bring the analysis of the composition of the gut microbiome one step closer to being part of routine health care analysis [55–57]. Thus, this method of detection for multiple clinically relevant microbial targets is a promising addition to current diagnostic techniques and treatment options.

## Supporting information

S1 Table Bioinformatics performance of the preliminary clinical target list. The 46 targets identified from literature and available clinical tests comprise 15 genera and 31 species. The bioinformatics pipeline for accurate detection of the maximum number of targets is optimized based on the performance metrics Sensitivity, Specificity, Precision and Negative Predictive Value (NPV) as determined with a manually curated amplicon database (described in S1 Doc). The metrics are calculated based on the number of true positives (TP), true negatives (TN), false positives (FP) and false negatives (FN) as follows: specificity = TN / (TN + FP), sensitivity = TP / (TP + FN), precision = TP / (TP + FP) and negative predictive value (NPV) = TN / (TN + FN).
(PDF)

S2 Table Health associations for each of the taxa on the screening test. All of the 28 taxa on the test have been associated with human health in the gut microbiome. This table has the associations for 13 specific conditions as identified in the scientific literature. Taxa are either associated or inversely associated. Microorganisms that associated with conditions have been shown to be elevated in patients suffering from these conditions. Microorganisms that are inversely associated were found to be less abundant in people who have this condition in the scientific literature.
(PDF)

S3 Table Relative abundances for the 28 clinical targets in fecal samples of 897 healthy individuals. A cohort of 897 self-reported healthy individuals from the uBiome microbiome research study was selected to define the healthy reference ranges for the relative abundances of 28 clinical targets in stool samples. The relative abundances for each target in each sample are presented as a percentage. The total number of reads in each sample is also noted.
(XLSX)

S4 Table Synthetic DNA sequences (sDNA) for the experimental validation. The following representative synthetic double-stranded DNA (sDNA) gene blocks were synthesized for the 28 taxa in the target list. These sDNA sequences were run through the clinical pipeline to validate accurate and quantitative detection.
(PDF)

S1 Doc Extended bioinformatics methodology. (PDF)

S2 Doc Accurate detection of all 28 targets. (PDF)
