# Correction: 16S rRNA gene sequencing and healthy reference ranges for 28 clinically relevant microbial taxa from the human gut microbiome

**DOI:** 10.1371/journal.pone.0212474

**Published:** 2019-02-12

**Authors:** Daniel E. Almonacid, Laurens Kraal, Francisco J. Ossandon, Yelena V. Budovskaya, Juan Pablo Cardenas, Elisabeth M. Bik, Audrey D. Goddard, Jessica Richman, Zachary S. Apte

There is an error in Fig 3. Specifically, the values for *Akkermansia muciniphila* and *Anaerotruncus colihominis* are swapped. A revised Fig 3 is provided here, which reflects the correct values as reported in S3 Table of the original article [1].

**Fig 3:**
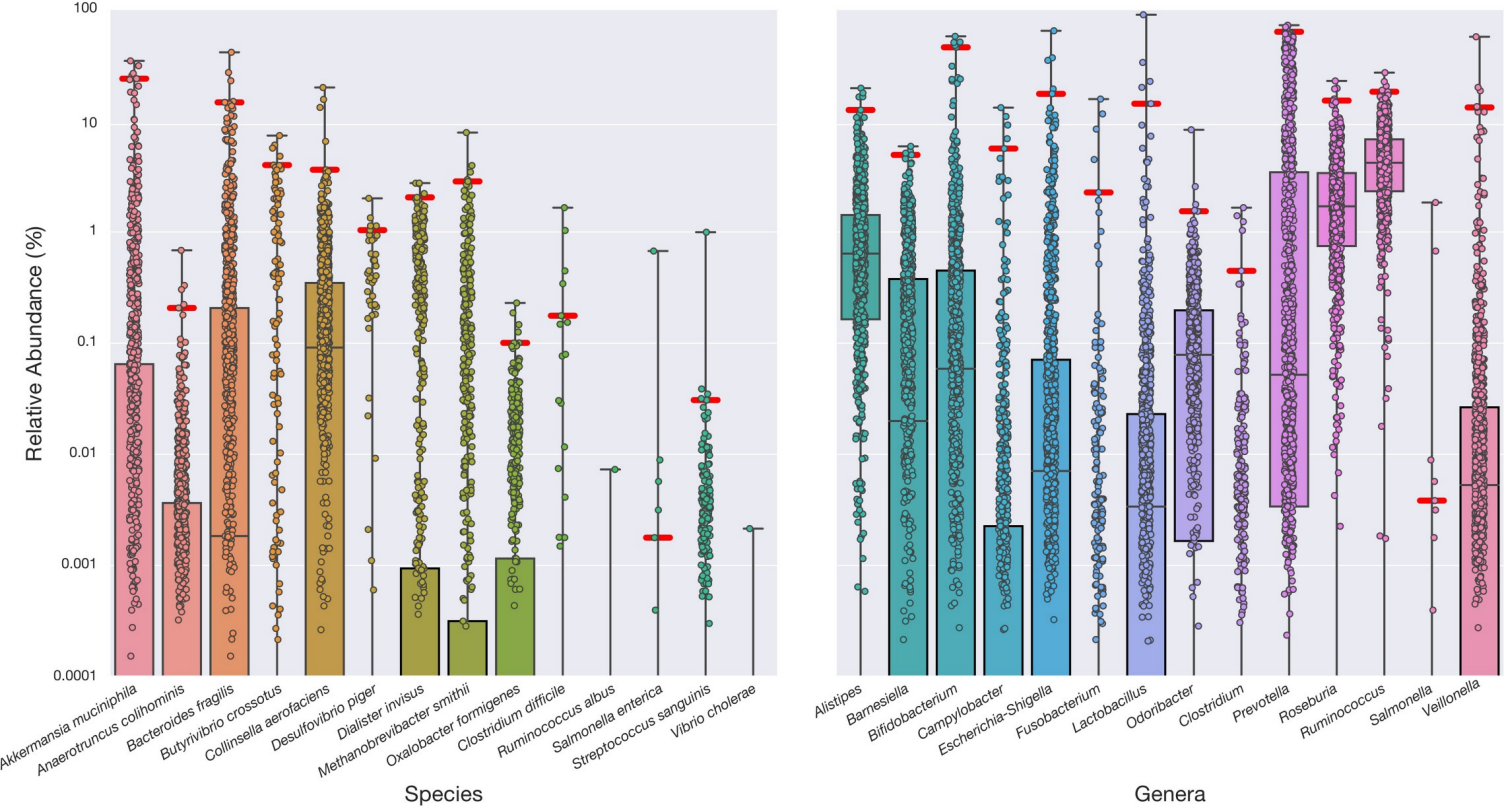
Reference ranges from a cohort of healthy individuals for 28 clinically relevant species and genera. Healthy participant stool microbiome data were analyzed to determine the empirical reference ranges for each target. The boxplot displays the relative abundance for each of 897 self-reported healthy individuals, revealing the healthy ranges of abundance for the taxa in the test panel. The healthy distribution is used to define the 99% confidence interval (red line). Boxes indicate the 25th–75th percentile, and the median coverage is indicated by a horizontal line in each box. Even in this healthy cohort, many of the bacteria that are associated with poor health conditions are present at some level. As most taxa are absent in a significant number of individuals most boxes expand to 0%, the healthy lower limit (not shown).

As stated in the Competing Interests statement on the original article, all authors have patents pending in relation to this work. The following sentences are added to the Competing Interests statement as an additional clarification: The healthy reference ranges reported in this article were used in the development of a commercially available test product developed and marketed by uBiome. This does not alter our adherence to PLOS ONE policies on sharing data and materials.
