# Expression of Concern: 16S rRNA gene sequencing and healthy reference ranges for 28 clinically relevant microbial taxa from the human gut microbiome

**DOI:** 10.1371/journal.pone.0276752

**Published:** 2022-10-20

**Authors:** The PLOS ONE Editors

Following publication of this article [1], concerns were raised about the origin of stool samples used to generate the microbiome dataset. Specifically, concerns were raised about the possible inclusion of samples from participants affected by health conditions or antibiotic usage, and non-humans.

The *PLOS ONE* Editors followed up with the authors, and it was confirmed that fecal samples were collected by participants, and those included in the study reported being healthy by survey; the study design did not exclude participants based on age. The reported age range for participants is 19 days– 103 years.

The first author has stated that an audit of customer service data held at uBiome in August 2019 identified information indicating that one of the samples submitted by a uBiome user, and labelled as a human gut sample, may have had a non-human origin (sample 757 as listed in S3 Table of [1]). This information was stored on a customer support system separate from the anonymized database used in the study and was unknown to the authors at the time of the study.

The first author provided the following additional information:

Evaluation of the data from the suspected non-human sample 757 indicates that this sample is not an outlier for the abundance for any microorganism. Recalculation of the values for the healthy ranges with sample 757 removed from the data set generates the same numerical values for the upper limit of the healthy range as those indicated by the red lines in Fig 3 of [1], previously corrected in [2]. Thus, because of the large sample size of this study, and the normal distribution of microorganism abundances in sample 757, inclusion or exclusion of sample 757 from the calculation of the healthy ranges does not alter the results and conclusions presented in the published article [1].

The corresponding author clarified that the uBiome user who submitted sample 757 had more than one sample in their account, which introduces the possibility that the non-human sample was not included in the study. They note that sample 757 clusters with the rest of the human samples in the study, which they say suggests a human origin for this sample.

Participants responded to a survey of 192 questions including items covering prebiotic, probiotic, and antibiotic use. The aggregate data from the full participant survey have not been provided. The corresponding author has stated that the study used data from six questions regarding age, gender, health, diagnosed medical conditions, diagnosed gut conditions, and symptoms at the time of sampling. A copy of those six questions is provided as Supporting Information (S1 File). Aggregate participant data for these six questions are provided as Supporting Information (S2 File).

The corresponding author may be contacted for scientific questions relating to the paper through the following updated email address: Zachary.apte@protonmail.com. The anonymized, individual-level participant data are restricted based on the terms of the consent forms and IRB approval. Following the closure of uBiome, the corresponding author has stated that the trustee of the company’s bankruptcy estate is responsible for retaining study data. The trustee’s contact details are as follows:

Alfred Giuliano

Trustee of uBiome Bankruptcy Estate

Giuliano, Miller, & Company

2301 E Evesham Road

800 Pavilion, Suite 210

Voorhees, NJ 08043

856.767.3000 ext. 11 phone

856.767.3500 fax

atgiuliano@giulianomiller.com

At the time of preparation of this notice, the *PLOS ONE* Editors have been unable to reach the named trustee to confirm whether the participant data underlying this study can be accessed upon suitable request.

The *PLOS ONE* Editors carried out an assessment of the published study in consultation with a member of the Editorial Board who advised that while the study provides data for the relative abundances of microbial taxa in the selected healthy cohort by 16S sequencing, it does not provide a basis to define clinically relevant relative levels of target microbial taxa and provides minimal data on the use of 16S sequencing for clinical diagnosis. It was additionally noted that the healthy range results reported from a self-selected sample of individuals interested in obtaining gut microbiome data may not be generalizable to other populations.

The *PLOS ONE* Editors issue this Expression of Concern to inform readers of the concerns about a possible non-human origin of one of the samples included in the study, and about the limitations of the study design with regard to drawing conclusions about clinically relevant levels of taxa and that routes to request access to restricted data do not appear to be functional at the time of preparation of this notice.

Additional methodological information has also been provided by the authors as follows:

The study design included participants of all ages and included 48 minors. Participation of minors aged 12 and below required permission from their legal guardian(s). Participation of minors 13 and above required assent of the conditions of the study and permission from their legal guardian(s). These methods of consent/assent and permission were reviewed and approved by the IRB (E&I Review Services, Missouri, IRB Study #13044, 05/10/2013).

The participants included in the study cohort were selected as 1000 samples meeting the criteria based on self-reported health status. The inclusion and exclusion criteria were selected with the aim of sampling a healthy cohort, rather than a representative cohort. Each participant had a unique password-protected participant account, to which the samples were associated. This association was used to ensure only one sample per user was selected for inclusion in the study, reducing the likelihood of duplicate samples.

Clear sampling instructions and a specially designed sampling kit were given to participants to reduce the likelihood of sampling error. Participants were strongly encouraged to register their sampling kit before use. Once registered, an online screen flow took the users step-by-step through sampling to reduce the likelihood of error. Samples were rejected on receipt in the laboratory if the sample was damaged in any way (e.g., it did not contain enough liquid or if it contained too much fecal matter to be processed). Collection and storage conditions were not expected to alter the outcome of this study as all samples were handled under identical laboratory conditions. The effects of sampling, storage and processing methods using uBiome collection methodology and buffers is reported in [3].

## Supporting information

S1 File Survey questions. **S**ix questions included in the 192-question survey regarding age, gender, health, diagnosed medical conditions, diagnosed gut conditions, and symptoms at the time of sampling.
(XLSX)

S2 File Aggregate participant data. (XLSX)
