# Supplementary material for: 16S rRNA gene sequencing and healthy reference ranges for 28 clinically relevant microbial taxa from the human gut microbiome
Source: PLoS One. 2017 May 3;12(5):e0176555. doi: 10.1371/journal.pone.0176555 (PMC5414997; doi:10.1371/journal.pone.0176555)
Supplement: S1 Table — The 46 targets identified from literature and available clinical tests comprise 15 genera and 31 species. The bioinformatics pipeline for accurate detection of the maximum number of targets is optimized based on the performance metrics Sensitivity, Specificity, Precision and Negative Predictive Value (NPV) as determined with a manually curated amplicon database (described in S1 Doc). The metrics are calculated based on the number of true positives (TP), true negatives (TN), false positives (FP) and false negatives (FN) as follows: specificity = TN / (TN + FP), sensitivity = TP / (TP + FN), precision = TP / (TP + FP) and negative predictive value (NPV) = TN / (TN + FN). (PDF) [file pone.0176555.s001.pdf]

Table S1
Bioinformatics performance of the preliminary clinical target list

The 46 targets identified from literature and available clinical tests comprise 15 genera and 31 species. The bioinformatics pipeline for accurate detection of the maximum number of targets is optimized based on the performance metrics Sensitivity, Specificity, Precision and Negative Predictive Value (NPV) as determined with a manually curated amplicon database (described in Doc S1). The metrics are calculated based on the number of true positives (TP), true negatives (TN), false positives (FP) and false negatives (FN) as follows:
specificity = TN / (TN + FP)
sensitivity = TP / (TP + FN)
precision = TP / (TP + FP)
negative predictive value (NPV) = TN / (TN + FN)

Genera

values in red are below our cut-off of 90%

Target	Sensitivity	Specificity	Precision	NPV
Alistipes	98.74	100.00	99.55	100.00
Barnesiella	99.32	100.00	99.73	100.00
Bifidobacterium	97.76	100.00	99.93	100.00
Campylobacter	98.60	100.00	99.90	100.00
Clostridium	93.05	100.00	96.58	99.99
Escherichia / Shigella	98.06	99.99	98.91	99.98
Fusobacterium	98.02	100.00	99.59	99.99
Lactobacillus	98.02	99.98	98.45	99.97
Odoribacter	98.87	100.00	99.86	100.00
Prevotella	98.63	100.00	99.88	100.00
Pseudoflavonifractor	15.38	100.00	100.00	100.00
Roseburia	91.34	99.97	91.85	99.97
Ruminococcus	94.97	99.99	95.45	99.99
Salmonella	97.09	100.00	98.69	100.00
Veillonella	99.07	100.00	99.97	100.00

Species

values in red are below our cut-off of 90%

Target	Sensitivity	Specificity	Precision	NPV
Akkermansia muciniphila	100.00	100.00	100.00	100.00
Anaerotruncus colihominis	100.00	100.00	100.00	100.00
Bacteroides fragilis	97.32	99.99	99.60	99.99
Bacteroides vulgatus	8.82	100.00	100.00	99.99
Bifidobacterium longum	10.11	100.00	100.00	99.99
Butyrivibrio crossotus	100.00	100.00	100.00	100.00
Campylobacter jejuni	6.73	100.00	100.00	99.97
Campylobacter coli	18.30	99.99	92.85	99.99
Campylobacter lari	7.93	100.00	100.00	99.99
Clostridium difficile	98.42	100.00	100.00	99.99
Collinsella aerofaciens	99.54	99.99	97.74	99.99
Coprococcus eutactus	50.00	100.00	100.00	99.99
Desulfovibrio piger	100.00	100.00	100.00	100.00
Dialister invisus	100.00	99.99	90.00	100.00
Escherichia coli	99.40	99.93	88.72	99.99
Escherichia coli O157	3.18	99.99	53.84	99.98
Faecalibacterium prausnitzii	16.66	100.00	100.00	99.99
Methanobrevibacter smithii	100.00	100.00	100.00	100.00
Oxalobacter formigenes	100.00	100.00	100.00	100.00
Ruminococcus albus	100.00	100.00	100.00	100.00
Ruminococcus bromii	20.00	100.00	100.00	99.99
Ruminococcus gnavus	20.00	99.99	66.66	99.99
Salmonella enterica	98.99	99.99	99.84	99.99
Salmonella bongori	4.16	100.00	100.00	99.99
Shigella boydii	14.46	99.99	30.26	99.99
Shigella sonnei	35.06	99.99	72.00	99.99
Shigella flexneri	12.03	99.99	35.25	99.97
Shigella dysenteriae	49.35	99.99	60.31	99.99
Streptococcus sanguinis	93.25	100.00	100.00	99.99
Streptococcus thermophilus	8.69	100.00	100.00	99.99
Vibrio cholerae	98.84	99.99	96.79	99.99
Yersinia enterocolitica	8.18	99.99	63.63	99.99
