# Supplementary material for: 16S rRNA gene sequencing and healthy reference ranges for 28 clinically relevant microbial taxa from the human gut microbiome
Source: PLoS One. 2017 May 3;12(5):e0176555. doi: 10.1371/journal.pone.0176555 (PMC5414997; doi:10.1371/journal.pone.0176555)
Supplement: S2 Table — All of the 28 taxa on the test have been associated with human health in the gut microbiome. This table has the associations for 13 specific conditions as identified in the scientific literature. Taxa are either associated or inversely associated. Microorganisms that associated with conditions have been shown to be elevated in patients suffering from these conditions. Microorganisms that are inversely associated were found to be less abundant in people who have this condition in the scientific literature. (PDF) [file pone.0176555.s002.pdf]

Table S2

Health associations for each of the taxa on the screening test

Associated: These taxa have been found to be more abundant in people who have this condition.

Inversely Associated: These taxa are found to be less abundant in people who have this condition.

Taxon	Associated	Inversely Associated
Akkermansia muciniphila	Type II Diabetes [30]	Obesity [39,40], Ulcerative Colitis [33], Crohn's disease [33]
Anaerotruncus colihominis	Bloating [41]	Obesity [1,42]
Bacteroides fragilis	Flatulence [43], Diarrhea [44,45]	
Butyrivibrio crossotus		Obesity [46,47]
Clostridium difficile	Diarrhea [48-50]	
Collinsella aerofaciens		IBS [4,51,52]
Desulfovibrio piger	IBD [53]	
Dialister invisus		Crohn's Disease [3]
Methanobrevibacter smithii	Abdominal Tenderness [54], Constipation [55]	Obesity [20,56]
Oxalobacter formigenes		Kidney Stones [57,58]
Ruminococcus albus		Ulcerative Colitis [59]
Salmonella enterica	IBS [5], Diarrhea [34-38]	
Streptococcus sanguinis	Kawasaki disease [60]	
Vibrio cholerae	Diarrhea [61,62]	
Alistipes		Obesity [1], IBS [1]
Barnesiella		Obesity [1]
Bifidobacterium		Constipation [2], Crohn’s Disease [3], IBS [4]
Campylobacter	IBS [5], Diarrhea [6,7]	
Clostridium	Diarrhea [8,9]	
Escherichia/Shigella	Crohn's Disease [10-12], IBS [5], Diarrhea [13-17]	
Fusobacterium	IBD [18]	
Lactobacillus	Obesity [19-21]	Obesity [20], Type II Diabetes [22,23], IBS [4,24], Diarrhea [25]
Odoribacter		Ulcerative Colitis [10], Crohn's Disease [10]
Prevotella		Ulcerative Colitis [26-28]
Roseburia		Type II Diabetes [29-31], Ulcerative Colitis [32], Crohn's Disease [27,28], IBD [10]
Ruminococcus	Ulcerative Colitis [33], Crohn's Disease [3,28,33]	
Salmonella	Diarrhea [34-38]	
Veillonella	IBS [24]	

References

1 Zhernakova A, Kurilshikov A, Bonder MJ, Tigchelaar EF, Schirmer M, Vatanen T, et al. Population-based metagenomics analysis reveals markers for gut microbiome composition and diversity. Science. 2016;352: 565–569. doi:10.1126/science.aad3369

2 Chassard C, Dapoigny M, Scott KP, Crouzet L, Del'homme C, Marquet P, et al. Functional dysbiosis within the gut microbiota of patients with constipated-irritable bowel syndrome. Aliment Pharmacol Ther. 2012;35: 828–838. doi:10.1111/j.1365-2036.2012.05007.x

3 Joossens M, Huys G, Cnockaert M, De Preter V, Verbeke K, Rutgeerts P, et al. Dysbiosis of the faecal microbiota in patients with Crohn's disease and their unaffected relatives. Gut. 2011;60: 631–637. doi:10.1136/gut.2010.223263

4 Malinen E. Association of symptoms with gastrointestinal microbiota in irritable bowel syndrome. World J Gastroenterol. 2010; 16: 4532–9. doi:10.3748/wjg.v16.i36.4532

5 Spiller RC. Role of infection in irritable bowel syndrome. J Gastroenterol. 2007;42 Suppl 17: 41–47. doi:10.1007/s00535-006-1925-8

6 Silva J, Leite D, Fernandes M, Mena C, Gibbs PA, Teixeira P. Campylobacter spp. as a Foodborne Pathogen: A Review. Front Microbio. Frontiers; 2011;2: 200. doi:10.3389/fmicb.2011.00200

7 Dasti JI, Tareen AM, Lugert R, Zautner AE, Groß U. Campylobacter jejuni A brief overview on pathogenicity-associated factors and disease-mediating mechanisms. International Jl of Medical Microbiology. Elsevier; 2010;300: 205–211. doi:10.1016/j.ijmm.2009.07.002

8 Brynestad S, Granum PE. Clostridium perfringens and foodborne infections. Int J Food Microbiol. 2002;74: 195–202.

9 Lund BM, Peck MW. Clostridium botulinum. Guide to Foodborne Pathogens. Oxford: John Wiley & Sons; 2013. pp. 91–111. doi:10.1002/9781118684856.ch6

10 Morgan XC, Tickle TL, Sokol H, Gevers D, Devaney KL, Ward DV, et al. Dysfunction of the intestinal microbiome in inflammatory bowel disease and treatment. Genome Biol. BioMed Central; 2012;13: R79. doi:10.1186/gb-2012-13-9-r79

11 Kang S, Denman SE, Morrison M, Yu Z, Doré J, Leclerc M, et al. Dysbiosis of fecal microbiota in Crohn's disease patients as revealed by a custom phylogenetic microarray. Inflamm Bowel Dis. 2010;16: 2034–2042. doi:10.1002/ibd.21319

12 Thorkildsen LT, Nwosu FC, Avershina E, Ricanek P, Perminow G, Brackmann S, et al. Dominant Fecal Microbiota in Newly Diagnosed Untreated Inflammatory Bowel Disease Patients. Gastroenterology Research and Practice. 2013;2013: 1–13. doi:10.1155/2013/636785

13 Liu D. Chapter 64. Diarrhoeagenic Escherichia coli. Molecular Medical Microbiology, Three-Volume Set. Elsevier Ltd; 2014. pp. 1133–1146. doi:10.1016/B978-0-12-397169-2.00064-0

14 Niyogi SK. Shigellosis. J Microbiol. 2005;43: 133–143.

15 Holmes LC. Shigella. Pediatr Rev. 2014;35: 261–262. doi:10.1542/pir.35-6-261

16 Anderson M, Sansonetti PJ, Marteyn BS. Shigella Diversity and Changing Landscape: Insights for the Twenty-First Century. Front Cell Infect Microbiol. 2016;6: a014159–9. doi:10.3389/fcimb.2016.00045

17 Lima IFN, Havt A, Lima AAM. Update on molecular epidemiology of Shigella infection. Curr Opin Gastroenterol. 2015;31: 30–37. doi:10.1097/MOG.0000000000000136

18 Strauss J, Kaplan GG, Beck PL, Rioux K, Panaccione R, DeVinney R, et al. Invasive potential of gut mucosa-derived fusobacterium nucleatum positively correlates with IBD status of the host. Inflamm Bowel Dis. 2011;17: 1971–1978. doi:10.1002/ibd.21606

19 Armougom F, Henry M, Vialettes B, Raccah D, Raoult D. Monitoring Bacterial Community of Human Gut Microbiota Reveals an Increase in Lactobacillus in Obese Patients and Methanogens in Anorexic Patients. Ratner AJ, editor. PLoS ONE. 2009;4: e7125–8. doi:10.1371/journal.pone.0007125

20 Million M, Maraninchi M, Henry M, Armougom F, Richet H, Carrieri P, et al. Obesity-associated gut microbiota is enriched in Lactobacillus reuteri and depleted in Bifidobacterium animalis and Methanobrevibacter smithii. Int J Obes (Lond). 2012;36: 817–825. doi:10.1038/ijo.2011.153

21 Million M, Angelakis E, Paul M, Armougom F, Leibovici L, Raoult D. Comparative meta-analysis of the effect of Lactobacillus species on weight gain in humans and animals. Microbial Pathogenesis. Elsevier Ltd; 2012;53: 100–108. doi:10.1016/j.micpath.2012.05.007

22 Ejtahed HS, Mohtadi-Nia J, Homayouni-Rad A, Niafar M, Asghari-Jafarabadi M, Mofid V. Probiotic yogurt improves antioxidant status in type 2 diabetic patients. Nutrition. Elsevier; 2012;28: 539–543. doi:10.1016/j.nut.2011.08.013

23 Tonucci LB, Santos dos KMO, de Oliveira LL, Ribeiro SMR, Martino HSD. Clinical application of probiotics in type 2 diabetes mellitus: A randomized, double-blind, placebo-controlled study. Clinical Nutrition. Elsevier Ltd; 2015;: 1–8. doi:10.1016/j.clnu.2015.11.011

24 Malinen E, Rinttila T, Kajander K, Matto J, Kassinen A, Krogius L, et al. Analysis of the Fecal Microbiota of Irritable Bowel Syndrome Patients and Healthy Controls with Real-Time PCR. Am J Gastroenterology. 2005;100: 373–382. doi:10.1111/j.1572-0241.2005.40312.x

25 Kale-Pradhan PB, Jassal HK, Wilhelm SM. Role of Lactobacillus in the prevention of antibiotic-associated diarrhea: a meta-analysis. Pharmacotherapy. Blackwell Publishing Ltd; 2010;30: 119–126. doi:10.1592/phco.30.2.119

26 Lepage P, Häsler R, Spehlmann ME, Rehman A, Zvirbliene A, Begun A, et al. Twin study indicates loss of interaction between microbiota and mucosa of patients with ulcerative colitis. Gastroenterology. Elsevier; 2011;141: 227–236. doi:10.1053/j.gastro.2011.04.011

27 Walters WA, Xu Z, Knight R. Meta-analyses of human gut microbes associated with obesity and IBD. FEBS Letters. Federation of European Biochemical Societies; 2014;588: 4223–4233. doi:10.1016/j.febslet.2014.09.039

28 Willing BP, Dicksved J, Halfvarson J, Andersson AF, Lucio M, Zheng Z, et al. A Pyrosequencing Study in Twins Shows That Gastrointestinal Microbial Profiles Vary With Inflammatory Bowel Disease Phenotypes. YGAST. Elsevier Inc; 2010;139: 1844–1854.e1. doi:10.1053/j.gastro.2010.08.049

29 Forslund K, Hildebrand F, Nielsen T, Falony G, Le Chatelier E, Sunagawa S, et al. Disentangling type 2 diabetes and metformin treatment signatures in the human gut microbiota. Nature. Nature Publishing Group; 2015;528: 262–266. doi:10.1038/nature15766

30 Qin J, Li Y, Cai Z, Li S, Zhu J, Zhang F, et al. A metagenome-wide association study of gut microbiota in type 2 diabetes. Nature. 2012;490: 55–60. doi:10.1038/nature11450

31 Larsen N, Vogensen FK, van den Berg FWJ, Nielsen DS, Andreasen AS, Pedersen BK, et al. Gut Microbiota in Human Adults with Type 2 Diabetes Differs from Non-Diabetic Adults. Bereswill S, editor. PLoS ONE. 2010;5: e9085–10. doi:10.1371/journal.pone.0009085

32 Machiels K, Joossens M, Sabino J, De Preter V, Arijs I, Eeckhaut V, et al. A decrease of the butyrate-producing species Roseburia hominis and Faecalibacterium prausnitzii defines dysbiosis in patients with ulcerative colitis. Gut. BMJ Publishing Group Ltd and British Society of Gastroenterology; 2014;63: 1275–1283. doi:10.1136/gutjnl-2013-304833

33 Png CW, Lindén SK, Gilshenan KS, Zoetendal EG, McSweeney CS, Sly LI, et al. Mucolytic bacteria with increased prevalence in IBD mucosa augment in vitro utilization of mucin by other bacteria. Am J Gastroenterology. 2010;105: 2420–2428. doi:10.1038/ajg.2010.281

34 Gal-Mor O, Boyle EC, Grassl GA. Same species, different diseases: how and why typhoidal and non-typhoidal Salmonella enterica serovars differ. Front Microbio. Frontiers; 2014;5: 391. doi:10.3389/fmicb.2014.00391

35 Guard-Petter J. The chicken, the egg and Salmonella enteritidis. Environ Microbiol. 2001;3: 421–430. doi:10.1128/IAI.02387-14

36 Wain J, Hendriksen RS, Mikoleit ML, Keddy KH, Ochiai RL. Typhoid fever. Lancet. 2015;385: 1136–1145. doi:10.1016/S0140-6736(13)62708-7

37 Santos RL. Chapter 72. Non-typhoidal Salmonella Interactions with Host Cells. Molecular Medical Microbiology, Three-Volume Set. Elsevier Ltd; 2014. pp. 1307–1318. doi:10.1016/B978-0-12-397169-2.00072-X

38 Sanderson KE, Liu S-L, Le Tang, Johnston RN. Chapter 71. Salmonella Typhi and Salmonella Paratyphi A. Molecular Medical Microbiology, Three-Volume Set. Elsevier Ltd; 2014. pp. 1275–1306. doi:10.1016/B978-0-12-397169-2.00071-8

39 Santacruz A, Collado MC, García-Valdés L, Segura MT, Martín-Lagos JA, Anjos T, et al. Gut microbiota composition is associated with body weight, weight gain and biochemical parameters in pregnant women. Br J Nutr. 2010;104: 83–92. doi:10.1017/S0007114510000176

40 Clarke SF, Murphy EF, O'Sullivan O, Lucey AJ, Humphreys M, Hogan A, et al. Exercise and associated dietary extremes impact on gut microbial diversity. Gut. BMJ Publishing Group Ltd and British Society of Gastroenterology; 2014;63: 1913–1920. doi:10.1136/gutjnl-2013-306541

41 Jalanka-Tuovinen J, Salonen A, Nikkilä J, Immonen O, Kekkonen R, Lahti L, et al. Intestinal Microbiota in Healthy Adults: Temporal Analysis Reveals Individual and Common Core and Relation to Intestinal Symptoms. Bereswill S, editor. PLoS ONE. 2011;6: e23035–13. doi:10.1371/journal.pone.0023035

42 Zupancic ML, Cantarel BL, Liu Z, Drabek EF, Ryan KA, Cirimotich S, et al. Analysis of the Gut Microbiota in the Old Order Amish and Its Relation to the Metabolic Syndrome. Thameem F, editor. PLoS ONE. 2012;7: e43052–10. doi:10.1371/journal.pone.0043052

43 Manichanh C, Eck A, Varela E, Roca J, Clemente JC, Gonzalez A, et al. Anal gas evacuation and colonic microbiota in patients with flatulence: effect of diet. Gut. 2014;63: 401–408. doi:10.1136/gutjnl-2012-303013

44 Durmaz B, Dalgalar M, Durmaz R. Prevalence of Enterotoxigenic Bacteroides fragilis in patients with diarrhea: A controlled study. Anaerobe. 2005;11: 318–321. doi:10.1016/j.anaerobe.2005.06.001

45 Keenan JI, Aitchison A, Purcell RV, Greenlees R, Pearson JF, Frizelle FA. Screening for enterotoxigenic Bacteroides fragilis in stool samples. Anaerobe. Elsevier Ltd; 2016;40: 50–53. doi:10.1016/j.anaerobe.2016.05.004

46 Le Chatelier E, Nielsen T, Qin J, Prifti E, Hildebrand F, Falony G, et al. Richness of human gut microbiome correlates with metabolic markers. Nature. 2013;500: 541–546. doi:10.1038/nature12506

47 Sze MA, Schloss PD. Looking for a Signal in the Noise: Revisiting Obesity and the Microbiome. mBio. 2016;7: e01018–16–9. doi:10.1128/mBio.01018-16

48 Surawicz CM, McFarland LV. Pseudomembranous colitis: causes and cures. Digestion. 1999;60: 91–100.

49 Surawicz CM, Brandt LJ, Binion DG, Ananthakrishnan AN, Curry SR, Gilligan PH, et al. Guidelines for Diagnosis, Treatment, and Prevention of Clostridium difficile Infections. Am J Gastroenterology. 2013;108: 478–498. doi:10.1038/ajg.2013.4

50 Ofosu A. Clostridium difficile infection: a review of current and emerging therapies. aog. 2016;29: 1–8. doi:10.20524/aog.2016.0006

51 Kassinen A, Krogius-Kurikka L, Mäkivuokko H, Rinttila T, Paulin L, Corander J, et al. The fecal microbiota of irritable bowel syndrome patients differs significantly from that of healthy subjects. YGAST. 2007;133: 24–33. doi:10.1053/j.gastro.2007.04.005

52 Jalanka-Tuovinen J, Salojärvi J, Salonen A, Immonen O, Garsed K, Kelly FM, et al. Faecal microbiota composition and host-microbe cross-talk following gastroenteritis and in postinfectious irritable bowel syndrome. Gut. BMJ Publishing Group Ltd and British Society of Gastroenterology; 2014;63: 1737–1745. doi:10.1136/gutjnl-2013-305994

53 Loubinoux J, Bronowicki J-P, Pereira IAC, Mougenel J-L, Faou AE. Sulfate-reducing bacteria in human feces and their association with inflammatory bowel diseases. FEMS Microbiology Ecology. 2002;40: 107–112. doi:10.1111/j.1574-6941.2002.tb00942.x

54 Weaver GA, Krause JA, Miller TL, Wolin MJ. Incidence of methanogenic bacteria in a sigmoidoscopy population: an association of methanogenic bacteria and diverticulosis. Gut. BMJ Group; 1986;27: 698–704.

55 Pimentel M, Mayer AG, Park S, Chow EJ, Hasan A, Kong Y. Methane production during lactulose breath test is associated with gastrointestinal disease presentation. Dig Dis Sci. 2003;48: 86–92.

56 Schwiertz A, Taras D, Schäfer K, Beijer S, Bos NA, Donus C, et al. Microbiota and SCFA in Lean and Overweight Healthy Subjects. Obesity. Nature Publishing Group; 2009;18: 190–195. doi:10.1038/oby.2009.167

57 Kaufman DW, Kelly JP, Curhan GC, Anderson TE, Dretler SP, Preminger GM, et al. Oxalobacter formigenes may reduce the risk of calcium oxalate kidney stones. J Am Soc Nephrol. 2008;19: 1197–1203. doi:10.1681/ASN.2007101058

58 Barnett C, Nazzal L, Goldfarb DS, Blaser MJ. The Presence of Oxalobacter formigenes in the Microbiome of Healthy Young Adults. Journal of Urology. Elsevier Ltd; 2016;195: 499–506. doi:10.1016/j.juro.2015.08.070

59 Fite A, Macfarlane S, Furrie E, Bahrami B, Cummings JH, Steinke DT, et al. Longitudinal Analyses of Gut Mucosal Microbiotas in Ulcerative Colitis in Relation to Patient Age and Disease Severity and Duration. J Clin Microbiol. 2013;51: 849–856. doi:10.1128/JCM.02574-12

60 Kinumaki A, Sekizuka T, Hamada H, Kato K, Yamashita A, Kuroda M. Characterization of the gut microbiota of Kawasaki disease patients by metagenomic analysis. Front Microbio. 2015;6: 4066–11. doi:10.3389/fmicb.2015.00824

61 Faruque SM, Albert MJ, Mekalanos JJ. Epidemiology, genetics, and ecology of toxigenic Vibrio cholerae. Microbiology and Molecular Biology Reviews. 1998;62: 1301–1314.

62 Harris JB, LaRocque RC, Qadri F, Ryan ET, Calderwood SB. Cholera. Lancet. 2012;379: 2466–2476. doi:10.1016/S0140-6736(12)60436-X
