# Supplementary material for: 16S rRNA gene sequencing and healthy reference ranges for 28 clinically relevant microbial taxa from the human gut microbiome
Source: PLoS One. 2017 May 3;12(5):e0176555. doi: 10.1371/journal.pone.0176555 (PMC5414997; doi:10.1371/journal.pone.0176555)
Supplement: S4 Table — The following representative synthetic double-stranded DNA (sDNA) gene blocks were synthesized for the 28 taxa in the target list. These sDNA sequences were run through the clinical pipeline to validate accurate and quantitative detection. (PDF) [file pone.0176555.s004.pdf]

Table S4

Synthetic DNA sequences (sDNA) for the experimental validation

The following representative synthetic double-stranded DNA (sDNA) gene blocks were synthesized for the 28 taxa in the target list. These sDNA sequences were run through the clinical pipeline to validate accurate and quantitative detection.

Target	sDNA sequence
Akkermansia muciniphila<br>SILVA ID:<br>AY271254.1.1433	TTCGGATTGTAAACCCCTGTCATGTGGGAGCAAATTAAAAAGATAGTACCACAAGAGGAAGAGAC<br>GGCTAACTCTGTGCCAGCAGCCGCGGTAATACAGAGGTCTCAAGCGTTGTTCGGAATCACTGGG<br>CGTAAAGCGTGCGTAGGCTGTTTCGTAAGTCGTGTGTGAAAGGCGCGGGCTCAACCCGCGGAC<br>GGCACATGATACTGCGAGACTAGAGTAATGGAGGGGGAACCGGAATTCTCGGTGTAGCAGTGAA<br>ATGCGTAGATATCGAGAGGAACACTCGTGGCGAAGGCGGGTTCCTGGACATTAACTGACGCTGA<br>GGCACGAAGGCCAGGGGAGCGAAAGGGATTAGATACCCCTGTAGTCCTGGCAGTAAACGGTGC<br>ACGCTTGGTGTGCGGGGAATCGACCCCCTGCGTGCCGGAGTAACGCGTTAAGCGTGCCG
Alistipes<br>SILVA ID:<br>AB554232.1.1488	TGCTTTTGTACGAGGGTAAACGCAGATACGTGTATCTGTCTGAAAGTATCGTACGAATAAGGATCG<br>GCTAACTCCGTGCCAGCAGCCGCGGTAATACGGAGGATTCAAGCGTTATCCGGATTTATTGGGTT<br>TAAAGGGTGCGTAGGCGGTTTGATAAGTTAGAGGTGAAATTTCGGGGCTCAACCCTGAACGTGC<br>CTCTAATACTGTTGAGCTAGAGAGTAGTTGCGGTAGGCGGAATGTATGGTGTAGCGGTGAAATGCT<br>TAGAGATCATACAGAACACCGATTGCGAAGGCAGCTTACCAAACTATATCTGACGTTGAGGCACG<br>AAAGCGTGGGGAGCAAACAGGATTAGATACCCTGGTAGTCCACGCAGTAAACGATGATAACTCGT<br>TGTCGGCGATACACAGTCGGTGACTAAGCGAAAGCGATAAGTTATCCACCT
Anaerotruncus colihominis<br>SILVA ID:<br>AJ315980.1.1393	CGGTCTTCGGATTGTAAACCTCTGTCTTTGGGGAAGAAAATGACGGTACCCAAAGAGGAAGCTC<br>CGGCTAACTACGTGCCAGCAGCCGCGGTAATACGTAGGGAGCAAGCGTTGTCCGGAATTACTGG<br>GTGTAAAGGGAGCGTAGGCGGGATGGCAAGTAGAATGTTAAATCCATCGGCTCAACCGGTGGCT<br>GCGTTCTAAACTGCCGTTCTTGAGTGAAGTAGAGGCAGGCGGAATTCCTAGTGTAGCGGTGAAAT<br>GCGTAGATATTAGGAGGAACACCAGTGGCGAAGGCGGCCTGCTGGGCTTTAACTGACGCTGAG<br>GCTCGAAAGCGTGGGGAGCAAACAGGATTAGATACCCTGGTAGTCCACGCCGTAAACGATGATTA<br>CTAGGTGTGGGGGGACTGACCCCTTCCGTGCCGCAGTTAACACAATAAGTAATCCA
Bacteroides fragilis<br>SILVA ID:<br>X83936.1.1436	TTCTTTTATATAAGAATAAAGTGCAGTATGTATACTGTTTTGTATGTATTATATGAATAAGGATCGGCTAA<br>CTCCGTGCCAGCAGCCGCGGTAATACGGAGGATCCGAGCGTTATCCGGATTTATTGGGTTTAAAG<br>GGAGCGTAGGTGGACTGGTAAGTCAGTTGTGAAAGTTTGCGGCTCAACCGTAAAATTGCAGTTGA<br>TACTGTCAGTCTTGAGTACAGTAGAGGTGGGCGGAATTCGTGGTGTAGCGGTGAAATGCTTAGATA<br>TCACGAAGAACTCCGATTGCGAAGGCAGCTCACTGGACTGCAACTGACACTGATGCTCGAAAGT<br>GTGGGTATCAAACAGGATTAGATACCCTGGTAGTCCACACAGTAAACGATGAATACTCGCTGTTTG<br>CGATATACAGTAAGCGGCCAAGCGAAAGCATTAAGTATTCCACCT

Barnesiella<br>SILVA ID:<br>AB370251.1.1484	CTCTTTTGTCGGAGAGTAAAGTACGCTACGTGTAGCGTATTGCAAGTATCCGAAGAAAAAGCATCG<br>GCTAACTCCGTGCCAGCAGCCGCGGTAATACGGAGGATGCGAGCGTTATCCGGATTTATTGGGTT<br>TAAAGGGTGCGTAGGCGGCACGCCAAGTCAGCGGTGAAATTTCCGGGCTCAACCCGGAGTGTG<br>CCGTTGAAACTGGCGAGCTAGAGTACACAAGAGGCAGGCGGAATGCGTGGTGTAGCGGTGAAA<br>TGCATAGATATCACGCAGAACCCCGATTGCGAAGGCAGCCTGCTAGGGTGAAACAGACGCTGAG<br>GCACGAAAGCGTGGGTATCGAACAGGATTAGATACCCTGGTAGTCCACGCAGTAAACGATGAATA<br>CTAACTGTTTGCGATACAATGTAAGCGGTACAGCGAAAGCGTTAAGTATTCCACCT
Bifidobacterium<br>SILVA ID:<br>AY305304.1.1448	GGTTGTAAACCGCTTTTGACTGGGAGCAAGCCCTTCGGGGTGAGTGTACCTTTCGAATAAGCAC<br>CGGCTAACTACGTGCCAGCAGCCGCGGTAATACGTAGGGTGCAAGCGTTATCCGGAATTATTGGG<br>CGTAAAGGGCTCGTAGGCGGTTCGTCGCGTCCGGTGTGAAAGTCCATCGCTTAACGGTGGATCC<br>GCGCCGGGTACGGGCGGGCTTGAGTGCGGTAGGGGAGACTGGAATTCCCGGTGTAACGGTGG<br>AATGTGTAGATATCGGGAAGAACACCAATGGCGAAGGCAGGTCTCTGGGCCGTCACTGACGCTG<br>AGGAGCGAAAGCGTGGGGAGCGAACAGGATTAGATACCCTGGTAGTCCACGCCGTAAACGGTG<br>GATGCTGGATGTGGGGACCATTCCACGGTCTCCGTGTCGGAGCCAACGCGTTAAGCATCC
Butyrivibrio crossotus<br>SILVA ID:<br>ABWN01000012.312.1822	AGTATTTCGGTATGTAAAGCTCTATCAGCAGGGAAGAAAACGACGGTACCTGACTAAGAAGCCCC<br>GGCTAACTACGTGCCAGCAGCCGCGGTAATACGTAGGGGGCAAGCGTTATCCGGATTTACTGGG<br>TGTAAAGGGAGCGTAGACGGCATCACAAGTCAGAAGTGAAAATCCGGGGCTCAACCCCGGAAC<br>TGCTTTTGAAACTGTGGAGCTGGAGTGCAGGAGAGGTAAGCGGAATTCCTAGTGTAGCGGTGAA<br>ATGCGTAGATATTAGGAGGAACACCAGTGGCGAAGGCGGCTTACTGGACTGTAACTGACGTTGA<br>GGCTCGAAAGCGTGGGGAGCAAACAGGATTAGATACCCTGGTAGTCCACGCCGTAAACGATGAA<br>TACTAGGTGTTGGGTTTCATAAGAAGCTCGGTGCCGGCGCAAACGCATTAAGTATTCC
Campylobacter<br>SILVA ID:<br>GU255922.1.1207	ACTTTTCGGAGCGTAAACTCCTTTTGTTAGGGAAGAACAATGACGGTACCTAACGAATAAGCACC<br>GGCTAACTCCGTGCCAGCAGCCGCGGTAATACGGAGGGTGCAAGCGTTACTCGGAATCACTGG<br>GCGTAAAGGACGCGTAGGCGGATTATCAAGTCTCTTGTGAAATCCTATGGCTTAACCATAGAACTG<br>CTTGGGAAACTGATAATCTAGAGTGAGGGAGAGGCAGATGGAATTGGTGGTGTAGGGGTAAAATC<br>CGTAGAGATCACCAGGAATACCCATTGCGAAGGCGATCTGCTGGAACTCAACTGACGCTAATGC<br>GTGAAAGCGTGGGGAGCAAACAGGATTAGATACCCTGGTAGTCCACGCCCTAAACGATGTATACT<br>AGTTGTTGCTAAGCTAGTCTTGGCAGTAATGCACCTAACGGATTAAGTATACCG
Collinsella aerofaciens<br>SILVA ID:<br>AJ245919.1.1432	CCGGCTAACTACGTGCCAGCAGCCGCGGTAATACGTAGGGGGCGAGCGTTATCCGGATTCATTG<br>GGCGTAAAGCGCGCGTAGGCGGCCCGGCAGGCCGGGGGTCGAAGCGGGGGGCTCAACCCC<br>CCGAAGCCCCCGGAACCTCCGCGGCTTGGGTCCGGTAGGGGAGGGTGGAACACCCGGTGTA<br>GCGGTGGAATGCGCAGATATCGGGTGGAACACCGGTGGCGAAGGCGGCCCTCTGGGCCGAGA<br>CCGACGCTGAGGCGCGAAAGCTGGGGGAGCGAACAGGATTAGATACCCTGGTAGTCCCAGCCG<br>TAAACGATGGACGCTAGGTGTGGGGGACGATCCCCCCGTGCCGCAGCCAACGCATTAAGCGTC<br>CCGCCT

Desulfovibrio piger<br>SILVA ID:<br>KF536745.1.1424	CAGAAGGGAAGAAACTAGGGTGTTCTAATCATCATCCTACTGACGGTACCTTCAAAGGAAGCACC<br>GGCTAACTCCGTGCCAGCAGCCGCGGTAATACGGAGGGTGCAAGCGTTAATCGGAATCACTGG<br>GCGTAAAGCGCACGTAGGCTGTTATGTAAGTCAGGGGTGAAAGCCCACGGCTCAACCGTGGAA<br>CTGCCCTTGATACTGCACGACTCGAATCCGGGAGAGGGTGGCGGAATTCCAGGTGTAGGAGTG<br>AAATCCGTAGATATCTGGAGGAACATCAGTGGCGAAGGCGGCCACCTGGACCGGTATTGACGCT<br>GAGGTGCGAAAGCGTGGGGAGCAAACAGGATTAGATACCCTGGTAGTCCACGCCGTAAACGATG<br>GATGCTAGATGTCGGGATGTATGTCTCGGTGTCGTAGTTAACGCGTTAAGCATCCCGCCT
Dialister invisus<br>SILVA ID:<br>JN713211.1.1560	ATCCGGGACGAGAAGGCAGGGTGCGAAGAACAAACTGCATTGACGGTACCGGAAAAGCAAGCC<br>ACGGCTAACTACGTGCCAGCAGCCGCGGTAATACGTAGGTGGCAAGCGTTGTCCGGAATTATTG<br>GGCGTAAAGCGCGCGCAGGCGGCTTCCCAAGTCCCTCTTAAAAGTGCGGGGCTTAACCCCGTG<br>ATGGAAAGGAAACTGGGAAGCTGGAGTATCGGAGAGGAAAGTGGAATTCCTAGTGTAGCGGTGA<br>AATGCGTAGAGATTAGGAAGAACACCGGTGGCGAAGGCGACTTTCTGGACGAAAACTGACGCTG<br>AGGCGCGAAAGCGTGGGGAGCAAACAGGATTAGATTCCCTGGTAGTCCACGCCGTAAACGATG<br>GATACTAGGTGTAGGAGGTATCGACCCCTCCTGTGCCGGAGTTAACGCAATAAGTATCCC
Escherichia-Shigella<br>SILVA ID:<br>GZ772340.7303.8824	CAGCGGGGAGGAAGGGAGTAAAGTTAATACCTTTGCTCATTGACGTTACCCGCAGAAGAAGCAC<br>CGGCTAACTCCGTGCCAGCAGCCGCGGTAATACGGAGGGTGCAAGCGTTAATCGGAATTACTGG<br>GCGTAAAGCGCACGCAGGCGGTTTGTTAAGTCAGATGTGAAATCCCCGGGCTCAACCTGGGAA<br>CTGCATCTGATACTGGCAAGCTTGAGTCTCGTAGAGGGGGGTAGAATTCCAGGTGTAGCGGTGA<br>AATGCGTAGAGATCTGGAGGAATACCGGTGGCGAAGGCGGCCCCCTGGACGAAGACTGACGCT<br>CAGGTGCGAAAGCGTGGGGAGCAAACAGGATTAGATACCCTGGTAGTCCACGCCGTAAACGATG<br>TCGACTTGGAGGTTGTGCCCTTGAGGCGTGGCTTCCGGAGCTAACGCGTTAAGTCGACCG
Fusobacterium<br>SILVA ID:<br>GQ301040.1.1415	TTTTCGGAATGTAAAGTGCTTTCAGTTGGGAAGAAAGAAATGACGGTACCAACAGAAGAAGTGAC<br>GGCTAAATACGTGCCAGCAGCCGCGGTAATACGTATGTCACGAGCGTTATCCGGATTTATTGGGC<br>GTAAAGCGCGTCTAGGTGGTTATGTAAGTCTGATGTGAAAATGCAGGGCTCAACTCTGTATTGCGT<br>TGGAAACTGTATAACTAGAGTACTGGAGAGGTAAGCGGAACTACAAGTGTAGAGGTGAAATTCGTA<br>GATATTTGTAGGAATGCCGATGGGGAAGCCAGCTTACTGGACAGATACTGACGCTGAAGCGCGAA<br>AGCGTGGGTAGCAAACAGGATTAGATACCCTGGTAGTCCACGCCGTAAACGATGATTACTAGGTG<br>TTGGGGGTCGAACCTCAGCGCCCAAGCAAACGCGATAAGTAATCCGCCT
Ruminococcus<br>SILVA ID:<br>X94967.1.1481<br>(The genus *Ruminococcus* corresponds to the sum of SILVA genera *Ruminococcus 1*, *Ruminococcus 2* and *Lachnoclostridium*)	AGTATTTCGGTATGTAAAGCTCTATCAGCAGGGAAGAAAATGACGGTACCTGACTAAGAAGCCCC<br>GGCTAACTACGTGCCAGCAGCCGCGGTAATACGTAGGGGGCAAGCGTTATCCGGATTTACTGGG<br>TGTAAAGGGAGCGTAGACGGCATGGCAAGCCAGATGTGAAAGCCCGGGGCTCAACCCCGGGA<br>CTGCATTTGGAACTGTCAGGCTAGAGTGTCGGAGAGGAAAGCGGAATTCCTAGTGTAGCGGTGA<br>AATGCGTAGATATTAGGAGGAACACCAGTGGCGAAGGCGGCTTTCTGGACGATGACTGACGTTG<br>AGGCTCGAAAGCGTGGGGAGCAAACAGGATTAGATACCCTGGTAGTCCACGCCGTAAACGATGA<br>ATACTAGGTGTCGGGTGGCAAAGCCATTCGGTGCCGCAGCAAACGCAATAAGTATTCCA

Lactobacillus<br>SILVA ID:<br>EF533990.1.1531	TGGTAGTGAAGAAAGATAGAGGTAGTAACTGGCCTTTATTTGACGGTAATTACTTAGAAAGTCACG<br>GCTAACTACGTGCCAGCAGCCGCGGTAATACGTAGGTGGCAAGCGTTGTCCGGATTTATTGGGC<br>GTAAAGCGAGTGCAGGCGGTTCAATAAGTCTGATGTGAAAGCCTTCGGCTCAACCGGAGAATTG<br>CATCAGAAACTGTTGAACTTGAGTGCAGAAGAGGAGAGTGGAACTCCATGTGTAGCGGTGGAAT<br>GCGTAGATATATGGAAGAACACCAGTGGCGAAGGCGGCTCTCTGGTCTGCAACTAACGCTGAGG<br>CTCGAAAGCATGGGTAGCGAACAGGATTAGATACCCTGGTAGTCCATGCCGTAAACGATGAGTGC<br>TAAGTGTTGGGAGGTTTCCGCCTCTCAGTGCTGCAGCTAACGCATTAAGCACTCC
Methanobrevibacter smithii<br>SILVA ID:<br>U55235.1.1357	TCCCAAGTGCCATTCTTAACGGGATGGCTTTTCATTAGTGTAAAGAGCTTTTGGAATAAGAGCTGG<br>GCAAGACCGGTGCCAGCCGCCGCGGTAACACCGGCAGCTCTAGTGGTAGCAGTTTTTATTGGG<br>CCTAAAGCGTCCGTAGCCGGTTTAATAAGTCTCTGGTGAAATCCTGCAGCTTAACTGTGGGAATTG<br>CTGGAGATACTATTAGACTTGAGATCGGGAGAGGTTAGAGGTACTCCCAGGGTAGAGGTGAAATT<br>CTGTAATCCTGGGAGGACCGCCTGTTGCGAAGGCGTCTGACTGGAACGATTCTGACGGTGAGG<br>GACGAAAGCTAGGGGCGCGAACCGGATTAGATACCCGGGTAGTCCTAGCTGTAAACGATGCGGA<br>CTTGGTGTTGGGGTGGCTTTGAGCTGTCCCAGTGCCGAAGGGAAGCTGTTAAGTCC
Odoribacter<br>SILVA ID:<br>AB490805.1.1478	CTCTTTTCTACTGGGAGAATAAGCCCTATGTATAAGGTGATGACAGTACAGTAGGAATAAGCATCGG<br>CTAACTCCGTGCCAGCAGCCGCGGTAATACGGAGGATGCGAGCGTTATCCGGATTTATTGGGTTT<br>AAAGGGTGCGTAGGCGGCTTTATAAGTTAGTGGTAAAATTTCGGAGCTTCACTCCGGTCCGCCAT<br>TAAAACTGTAGAGCTAGAGAATGGACGAGGTAGGCGGAATAAGTTAAGTAGCGGTGAAATGCATAG<br>ATATAACTTAGAACTCCGATAGCGAAGGCAGCTTACCAGACCATAACTGACGCTGATGCACGAGA<br>GCGTGGGTAGCGAACAGGATTAGATACCCTGGTAGTCCACGCCGTAAACGATGCTCACCGGCCC<br>TTAGCGATAAGACAGTTAGGGGTTAATTGAAAGAATTAAGTGAGCCACC
Oxalobacter formigenes<br>SILVA ID:<br>U49757.1.1488	TGTCAGGGAAGAAATTGATTGGGCTAATATCCCGATTAGATGACGGTACCTGAAGAATAAGCACCG<br>GCTAACTACGTGCCAGCAGCCGCGGTAATACGTAGGGTGCGAGCGTTAATCGGAATTACTGGGC<br>GTAAAGCGTGCGCAGGCGGTTGTGTAAGACAGATGTGAAATGCCCGGGCTCAACCTGGGAATTG<br>CATTTGTGACTGCACGGCTAGAGTGTGTCAGAGGGGGGTAGAATTCCACGTGTAGCAGTGAAAT<br>GCGTAGATATGTGGAGGAATACCGATGGCGAAGGCAGCCCCCTGGGATAACACTGACGCTCATG<br>CACGAAAGCGTGGGGAGCAAACAGGATTAGATACCCTGGTAGTCCACGCACTAAACGATGTCGA<br>CTAGTTGTTGGGTCTTAATAGACTTAGTAACGCAGCTAACGCGTGAAGTCGACCGC
Clostridium<br>SILVA ID:<br>AB023971.1.1404<br>(The genus *Clostridium* corresponds to SILVA genus *Peptoclostridium*)	AGGCCTTCGGGTCGTAAAGCTCTGTCCTCAAGGAAGATAATGACGGTACTTGAGGAGGAAGCCC<br>CGGCTAACTACGTGCCAGCAGCCGCGGTAATACGTAGGGGGCTAGCGTTATCCGGATTTACTGG<br>GCGTAAAGGGTGCGTAGGCGGTCTTTCAAGTCAGGAGTTAAAGGCTACGGCTCAACCGTAGTAA<br>GCTCCTGATACTGTCTGACTTGAGTGCAGGAGAGGAAAGCGGAATTCCCAGTGTAGCGGTGAAA<br>TGCGTAGATATTGGGAGGAACACCAGTAGCGAAGGCGGCTTTCTGGACTGTAACTGACGCTGAG<br>GCACGAAAGCGTGGGGAGCAAACAGGATTAGATACCCTGGTAGTCCACGCTGTAAACGATGAGT<br>ACTAGGTGTCGGAGGTTACCCCTTCGGTGCCGCAGCTAACGCATTAAGTACTCCGCC

Clostridium difficile<br>SILVA ID:<br>FN665653.3773870.3775372<br>(The species *Clostridium difficile* corresponds to SILVA species *Peptoclostridium difficile*)	AGGCCTTCGGGTCGTAAAACTCTGTCCTCAAGGAAGATAATGACGGTACTTGAGGAGGAAGCCC<br>CGGCTAACTACGTGCCAGCAGCCGCGGTAATACGTAGGGGGCTAGCGTTATCCGGATTTACTGG<br>GCGTAAAGGGTGCGTAGGCGGTCTTTCAAGTCAGGAGTGAAAGGCTACGGCTCAACCGTATTAA<br>GCTCTTGAAACTGGGAGACTTGAGTGCAGGAGAGGAGAGTGGAATTCCTAGTGTAGCGGTGAAA<br>TGCGTAGATATTAGGAGGAACACCAGTTGCGAAGGCGGCTCTCTGGACTGTAACTGACGCTGAG<br>GCACGAAAGCGTGGGGAGCAAACAGGATTAGATACCCTGGTAGTCCACGCTGTAAACGATGAGT<br>ACTAGGTGTCGGGGGTTACCCCCCTCGGTGCCGCAGCTAACGCATTAAGTACTCCGC
Prevotella<br>SILVA ID:<br>AB547700.1.1481<br>(The genus *Prevotella* corresponds to the sum of SILVA genera *Prevotella*, *Prevotella 1*, *Prevotella 2*, *Prevotella 6*, *Prevotella 7* and *Prevotella 9*)	TGCTTTTATGCGGGGATAAAGTGAGGGACGTGTCCTTCATTGCAGGTACCGCATGAATAAGGACC<br>GGCTAATTCCGTGCCAGCAGCCGCGGTAATACGGAAGGTCCGGGCGTTATCCGGATTTATTGGGT<br>TTAAAGGGAGCGTAGGCCGTGGATTAAGCGTGTTGTGAAATGCAGGTGCTCAACGTCTGCACTG<br>CAGCGCGAACTGGTCCACTTGAGTGTGCGCAACGCAGGCGGAATTCGTCGTGTAGCGGTGAAA<br>TGCTTAGATATGACGAAGAACTCCGATTGCGAAGGCAGCTTGCGGGAGCACAACTGACGCTGAA<br>GCTCGAAAGTGCGGGTATCGAACAGGATTAGATACCCTGGTAGTCCGCACGGTAAACGATGGATG<br>CCCGTTGTCAGGCTGTTTCAGCCTGGTGACCAAGCGAAAGCATTAAGCATCCCACC
Roseburia<br>SILVA ID:<br>AJ270473.1.1454	TATTTCGGTATGTAAAGCTCTATCAGCAGGGAAGAAGAAATGACGGTACCTGACTAAGAAGCACCG<br>GCTAAATACGTGCCAGCAGCCGCGGTAATACGTATGGTGCAAGCGTTATCCGGATTTACTGGGTGT<br>AAAGGGAGCGCAGGCGGAAGGCTAAGTCTGATGTGAAAGCCCGGGGCTCAACCCCGGTACTG<br>CATTGGAAACTGGTCATCTAGAGTGTCGGAGGGGTAAGTGGAATTCCTAGTGTAGCGGTGAAATG<br>CGTAGATATTAGGAGGAACACCAGTGGCGAAGGCGGCTTACTGGACGATAACTGACGCTGAGGC<br>TCGAAAGCGTGGGGAGCAAACAGGATTAGATACCCTGGTAGTCCACGCCGTAAACGATGAATACT<br>AGGTGTCGGAAAGCACAGCTTTTCGGTGCCGCCGCAAACGCATTAAGTATTCCA
Ruminococcus albus<br>SILVA ID:<br>X85098.1.1458	AGGTTTTAGGATTGTAAACCTCTGTCTTTGGGGACGATAATGACGGTACCCAAGGAGGAAGCTCC<br>GGCTAACTACGTGCCAGCAGCCGCGGTAATACGTAGGGAGCGAGCGTTGTCCGGAATTACTGGG<br>TGTAAAGGGAGCGTAGGCGGGATTGCAAGTCAGGTGTGAAATTTAGGGGCTTAACCCCTGAACT<br>GCACTTGAAACTGTAGTTCTTGAGTGAAGTAGAGGTAAGCGGAATTCCTAGTGTAGCGGTGAAAT<br>GCGTAGATATTAGGAGGAACATCAGTGGCGAAGGCGGCTTACTGGGCTTTAACTGACGCTGAGG<br>CTCGAAAGCGTGGGGAGCAAACAGGATTAGATACCCTGGTAGTCCACGCCGTAAACGATGATTAC<br>TAGGTGTGGGGGGACTGACCCCTTCCGTGCGCAGTTAACACAATAAGTAATCCAC
Salmonella<br>SILVA ID:<br>AF029226.1.1498	CAGCGGGGAGGAAGGCGATAAGGTTAATAACCTTGTTGATTGACGTTACCCGCAGAAGAAGCAC<br>CGGCTAACTCCGTGCCAGCAGCCGCGGTAATACGGAGGGTGCAAGCGTTAATCGGAATTACTGG<br>GCGTAAAGCGCACGCAGGCGGTCTGTCAAGTCGGATGTGAAATCCCCGGGCTGAACCTGGGAA<br>CTGCATCCGAAACTGGCAAGCTTGAGTCTCGTAGAGGGGGGTAGAATTCCAGGTGTAGCGGTGA<br>AATGCGTAGAGATCTGGAGGAATACCGGTGGCGAAGGCGGCCCCCTGGACGAAGACTGACGCT<br>CAGGTGCGAAAGGGTGGGGAGCAAACAGGATTAGATACCCTGGTAGTCCACGCCGTAAACGATG<br>TCGACTTGGAGGTTGTGCCCTTGAGGCGTGGCTTCCGGAGCTAACGCGTTAAGTCGACCG

Salmonella enterica<br>SILVA ID:<br>AB273733.1.1331	CAGCGGGGAGGAAGGTGTTGTGGTTAATAACCGCAGCAATTGACGTTACCCGCAGAAGAAGCAC<br>CGGCTAACTCCGTGCCAGCAGCCGCGGTAATACGGAGGGTGCAAGCGTTAATCGGAATTACTGG<br>GCGTAAAGCGCACGCAGGCGGTCTGTCAAGTCGGATGTGAAATCCCCGGGCTCAACCTGGGAA<br>CTGCATTCGAAACTGGCAGGCTTGAGTCTTGTAGAGGGGGGTAGAATTCCAGGTGTAGCGGTGA<br>AATGCGTAGAGATCTGGAGGAATACCGGTGGCGAAGGCGGCCCCCTGGACAAAGACTGACGCT<br>CAGGTGCGAAAGCGTGGGGAGCAAACAGGATTAGATACCCTGGTAGTCCACGCCGTAAACGATG<br>TCTACTTGGAGGCTGTGCCCTTGAGGCGTGGCTTCCGGAGCTAACGCGTTAAGTAGACCG
Streptococcus sanguinis<br>SILVA ID:<br>AB002524.1.1358	GTAAGAGAAGAACGGGTGTGAGAGTGGAAAGTTCACACTGTGACGGTATCTTACCAGAAAGGGA<br>CGGCTAACTACGTGCCAGCAGCCGCGGTAATACGTAGGTCCCGAGCGTTGTCCGGATTTATTGG<br>GCGTAAAGCGAGCGCAGGCGGTTAGATAAGTCTGAAGTTAAAGGCTGTGGCTTAACCATAGTATG<br>CTTTGGAAACTGTTTAACTTGAGTGCAGAAGGGGAGAGTGGAATTCCATGTGTAGCGGTGAAATG<br>CGTAGATATATGGAGGAACACCGGTGGCGAAAGCGGCTCTCTGGTCTGTAACTGACGCTGAGGC<br>TCGAAAGCGTGGGGAGCAAACAGGATTAGATACCCTTGTAGTCCACGCCGTAAACGATGAGTGCT<br>AGGTGTTAGGCCCTTTCCGGGGCTCAGTGCCGCAGCTAACGCATTAAGCACTCC
Veillonella<br>SILVA ID:<br>X84006.1.1513	AATCGGGACGAAAGGCCTTCTTGCGAATAGTTAGAAGGATTGACGGTACCGGAATAGAAAGCCAC<br>GGCTAACTACGTGCCAGCAGCCGCGGTAATACGTAGGTGGCAAGCGTTGTCCGGAATTATTGGG<br>CGTAAAGCGCGCGCAGGCGGATTGGTCAGTCTGTCTTAAAAGTTCGGGGCTTAACCCCGTGATG<br>GGATGGAAACTGCCAATCTAGAGTATCGGAGAGGAAAGTGGAATTCCTAGTGTAGCGGTGAAATG<br>CGTAGATATTAGGAAGAACACCAGTGGCGAAGGCGACTTTCTGGACGAAAACTGACGCTGAGGC<br>GCGAAAGCCAGGGGAGCGAACGGGATTAGATACCCCGGTAGTCCTGGCCGTAAACGATGGGTA<br>CTAGGTGTAGGAGGTATCGACCCCTTCTGTGCCGGAGTTAACGCAATAAGTACCCC
Vibrio cholerae<br>SILVA ID:<br>AY292952.1.1451	CAGTAGGGAGGAAGGTGGTTAAGTTAATACCTTAATCATTTGACGTTACCTACAGAAGAAGCACCG<br>GCTAACTCCGTGCCAGCAGCCGCGGTAATACGGAGGGTGCAAGCGTTAATCGGAATTACTGGGC<br>GTAAAGCGCATGCAGGTGGTTTGTTAAGTCAGATGTGAAAGCCCTGGGCTCAACCTAGGAATCG<br>CATTTGAAACTGACAAGCTAGAGTACTGTAGAGGGGGGTAGAATTTCAGGTGTAGCGGTGAAATG<br>CGTAGAGATCTGAAGGAATACCGGTGGCGAAGGCGGCCCCCTGGACAGATACTGACACTCAGAT<br>GCGAAAGCGTGGGGAGCAAACAGGATTAGATACCCTGGTAGTCCACGCCGTAAACGATGTCTAC<br>TTGGAGGTTGTGCCCTAGAGGTGTGGCTTTCGGAGCTAACGCGTTAAGTAGACCG
