# Supplementary material for: 16S rRNA gene sequencing and healthy reference ranges for 28 clinically relevant microbial taxa from the human gut microbiome
Source: PLoS One. 2017 May 3;12(5):e0176555. doi: 10.1371/journal.pone.0176555 (PMC5414997; doi:10.1371/journal.pone.0176555)
Supplement: S1 Doc — (PDF) [file pone.0176555.s005.pdf]

Document S1

Extended bioinformatics methodology

Taxonomic database generation

To generate the taxonomic database used to implement the clinical test that we present, we first predicted the amplicons that would be produced by V4 primers for all the sequences in the SILVA database. The primers used were GTGCCAGCMGCCGCGGTAA (forward) and GGACTACHVGGGTWTCTAAT (reverse), where M is A or C, H is A, C or T, V is A, C or G and W is A or T. We allowed annealing with up to 2 mismatches. The resulting predicted amplicons were subsequently inspected for degenerate bases. Degenerate amplicons that expanded to more than 20 possible non-degenerate sequences were regarded as bad quality sequences and eliminated from the database, whereas those that expanded to less than 20 possible sequences were kept expanded as each of their non-degenerate sequences. The total bacterial and archaeal species that passed the filters above are 1,611,503 unique sequences corresponding to 1,728,313 non-degenerate sequences. Given our use of pair-end sequencing, we further processed the amplicons, so that they are represented by a forward read containing the forward primer and 125bp to the 3' end of the forward primer, and a reverse read containing the reverse primer and 124bp to the 3' end of the reverse primer. Finally, primers were removed, and the remainder of the reads (125bp after the forward primer plus +124bp after the primer read) were concatenated and stored in an amplicon database.

The 1,611,503 amplicons for all the sequences in the SILVA database correspond to 5659 genera and 153619 species, meaning that there are various amplicon sequences for the same taxa. These sequences represent the known biological variations of the rRNA gene per taxa that results of the natural divergent evolution of DNA sequences. Some of these amplicons are unspecific to one taxa and may match various different taxa, which may be the result of this natural divergent evolution, but also misannotation. By selectively removing subsets of unspecific amplicons for each taxa, several curated databases are created per taxa, and the best database identified using the procedures outlined below. Our taxonomy annotation is based on sequence similarity searches of pair-end reads using 100% identity over 100% of the length against the set of sequences in these curated databases. The sequences present in a curated database for each species or genus are what define the elements of the confusion matrices and therefore the performance metrics for predictions. By excluding unspecific amplicons from consideration the number of false positives is reduced at the expense of the identification of true positives. To optimize this trade-off we first identified the true positives and all their identical sequences and then generated databases that include varying amounts of unspecific sequences. The sequences that are unambiguously annotated to a taxa were included first. Then, sequences that were

annotated to the taxa of interest (ti), but also to a different taxa (dt) were ranked according to the quotient dt/ti and were included in groups according to the values of their quotient. On one extreme, for example, when the quotient is 0, we created very specific databases where no false positives were allowed. At the other extreme, when the quotient is large, 100 for example, we created very sensitive databases where we maximized the identification of true positives, at the expense of generating false positives. We extensively explored different possible databases between these two extremes by using each of the generated databases to predict the taxonomy of all 16S sequences for each of the species and genera of interest. We computed confusion matrices and determined performance metrics for prediction and then explored the performance metrics for predictions for the different databases of the same species or genus. In other words, we selectively removed sequences with unspecific amplicons from the database while maximizing the sensitivity, specificity, precision and negative predictive value of identification for the majority of the sequences in each taxonomic group. We selected as the best databases for each taxa those where sensitivity, specificity, precision and negative predictive value were all above 90%, where the distance between precision and specificity is the minimum possible value, and aimed at favoring precision over specificity whenever possible.
