# Supplementary material for: 16S rRNA gene sequencing and healthy reference ranges for 28 clinically relevant microbial taxa from the human gut microbiome
Source: PLoS One. 2017 May 3;12(5):e0176555. doi: 10.1371/journal.pone.0176555 (PMC5414997; doi:10.1371/journal.pone.0176555)
Supplement: S2 Doc — (PDF) [file pone.0176555.s006.pdf]

Document S2

Accurate detection of all 28 targets

To demonstrate our ability to accurately detect all the microorganisms in the clinical target list, we created representative synthetic double-stranded DNA (sDNA) gene blocks for each of the 28 targets (Fig S1, S4 Table). We analyzed the 28 targets as two sets of 14 distinct sDNA sequences. These sDNA sets were combined in specific proportions, resulting in five samples of increasing ratios for each target, 1:14,014, 1:1,414, 1:714 1:154 and ~1:14. We processed each sample using our clinical bioinformatics pipeline. Importantly, we accurately detected all targets at each ratio (Fig S2), except at the 1:14,014 ratio where the genus Escherichia-Shigella was not identified. At all other ratios, the average abundance of targets was within 2.9 times of the predicted average abundance. Thus, from this experiment we conclude that our limit of detection is at the 0.0707% predicted average abundance, which corresponds to an experimental average abundance of 0.032%. Our theoretical limit of detection, determined by our sequencing depth of at least 10,000 reads per sample and by the fact that we only annotate targets for which there is at least two reads in a sample, is 2:10,000 or 0.02%. Thus, experimental results are in accordance to what was expected from theory. Our ability to accurately determine the relative abundance of each target, even when it is present at exceedingly low levels, suggests that we can accurately detect each target within clinical samples and relate it to the healthy reference range to obtain a clinically informative result.

Method

Double-stranded DNA segments were designed to be representative for the V4 region of the 16S rRNA gene of each target species or genus and synthesized by IDT and Thermo Fisher. Two sets of 14 targets each were combined at 1:10, 1:50, 1:100 and 1:1000 ratios and vice versa, allowing for the detection of different levels of targets in a high background of DNA (the undiluted set). The resulting ratios are 1:14014, 1:1414, 1:714, 1:154, and ~1:14 (1000:14014 for the undiluted set) for each individual target (Fig S1). The amount of DNA for each target was 1.74 pg (6.078 attomoles) before dilution. Sample combinations were processed in uBiome microbiome sampling kits using the clinical pipeline described in the main manuscript.

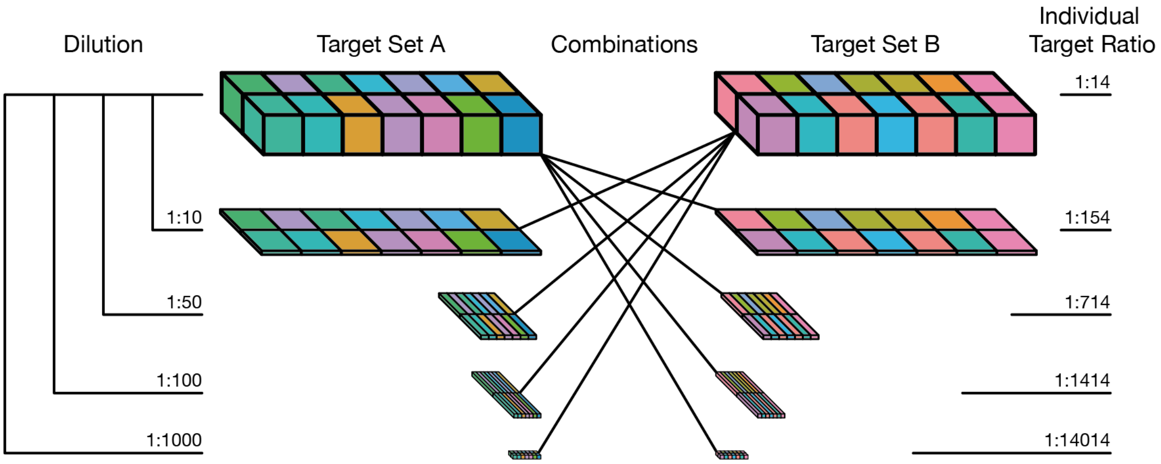

Fig S1. Target sDNA set combinations.

The 28 targets were combined as two sets of 14 distinct sDNA sequences, target set A and target set B. Both of these sDNA sets were diluted and combined with the undiluted set in specific proportions (1:10, 1:50, 1:100 and 1:1000). Four dilutions of target set A were combined with the undiluted target set B and vice versa. The resulting ratio for each individual target in the diluted set is 1:154, 1:714, 1:1414 and 1:14014. The ratio of the individual targets in the undiluted set is 1:14.

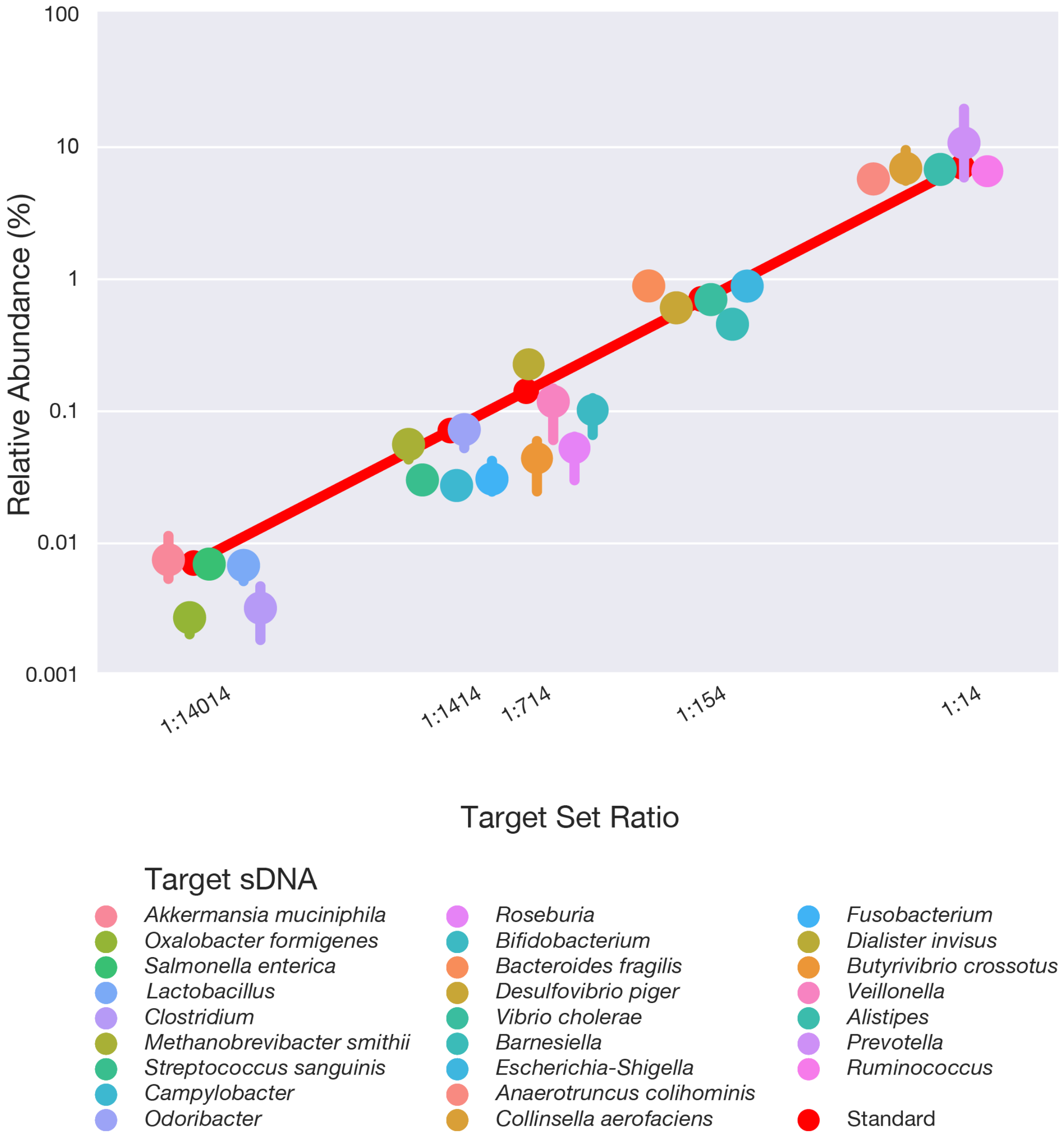

Fig S2. Experimental validation of the clinical 16S rRNA gene sequencing for the 28 targets on the test panel using synthetic DNA.

Targets in the test panel are readily detected at various levels of relative abundance. Double-stranded DNA segments were designed to be representative for the V4 region of the 16S rRNA gene of each target species or genus. Two sets of 14 targets each were combined in 1:10, 1:50, 1:100 and 1:1000 ratios and vice versa, resulting in the following expected relative abundances of each target in the diluted set: 0.65%, 0.14%, 0.071% and 0.0071%. The abundance of the undiluted set in these ratios is plotted as 1:1 at an expected 7.1%. The expected abundances are plotted in red, while the average abundance of 5 representative targets is plotted with a confidence interval. The plot shows that targets are detected at different levels of relative abundance in the test panel within a high background of DNA.
